# Multifunctional anionic nanoemulsion with linseed oil and lecithin: a preliminary approach for dry eye disease

**DOI:** 10.3762/bjnano.16.120

**Published:** 2025-10-02

**Authors:** Niédja Fittipaldi Vasconcelos, Almerinda Agrelli, Rayane Cristine Santos da Silva, Carina Lucena Mendes-Marques, Isabel Renata de Souza Arruda, Priscilla Stela Santana de Oliveira, Mércia Liane de Oliveira, Giovanna Machado

**Affiliations:** 1 Centro de Tecnologias Estratégicas do Nordeste (CETENE), Avenida Professor Luiz Freire 01, Cidade Universitária, 50740-540, Recife, Pernambuco, Brazil

**Keywords:** eye drops, micelles, low-energy method, ophthalmic vehicle, sample dilution, stability

## Abstract

The treatment of dry eye disease (DED) often requires frequent use of artificial tear products. Because of their low permeability and limited ocular bioavailability, repeated applications are required for therapeutic effectiveness. In contrast to traditional drug delivery systems (DDS), a functional ophthalmic nanoemulsion was specifically designed to alleviate symptoms of DED by leveraging its antioxidant and osmoprotective properties. The study evaluated the optimal concentration of lecithin required to produce nanoemulsions with a uniform particle size and incorporated a co-surfactant to enhance the stability of the nanoformulation. A straightforward method was proposed, involving the dilution of the preformulation in an ophthalmic vehicle, followed by homogenization through ultrasonication, resulting in OphtNE-3.70% with a droplet diameter of 173 nm and a zeta potential of −44.7 mV. The addition of Kolliphor^®^ HS15 in OphtNE-3.66%(K1%) initially reduced the droplet size to 70.8 nm and enhanced the antioxidant effect. Although the droplet size and polydispersity index increased after more than 60 days, the formulation remained physically quite stable without phase separation. Both nanoformulations contained 2.6% (w/v) linseed oil, providing a bioactive concentration compatible with ocular administration volumes (~50 µL). At a final concentration of 1.30 mg·mL^−1^, OphtNE-3.66%(K1%) showed >75% cell viability in L929 cells and ~10% 2,2-diphenyl-1-picrylhydrazyl (DPPH) antioxidant effect. These findings support the multifunctional potential (cytocompatibility and antioxidant) of sterile OphtNE-3.66%(K1%) for the treatment of DED, emphasizing the need for in vivo studies to ensure its efficacy and safety for ocular administration.

## Introduction

Dry eye disease (DED) is a multifactorial condition affecting the ocular surface, characterized by changes in tear fluid composition and/or insufficient tear production [1]. This condition can cause ocular discomfort, impair visual function, and promote inflammatory processes on the ocular surface, which could result in chronic complications and vision loss [2–3]. DED affects approximately 11.6% of the global population [4], with this prevalence partly increasing due to the widespread use of screens and computers [5].

Systemic administration of medications for treating ocular diseases requires high doses to achieve therapeutic effects, which can increase the risk of toxicity [6]. Several barriers limit the penetration of ocular medications, such as rapid elimination by the precorneal layer and low retention of the ocular surface [7]. As a result, topical administration remains the primary treatment method despite its drawbacks, such as low bioavailability and the need for frequent applications [8–9]. Studies show that only a tiny fraction (approximately 5% or 1.5 µL) of the administered dose reaches the ocular chamber after topical application to the inferior conjunctival sac [10–11]. The topical use of artificial tears is the standard treatment for symptomatic relief from DED [12], while topical anti-inflammatory therapy is used in chronic disease cases [13]. Advances in nanomedicine have provided effective solutions, particularly for treating DED [1].

To address challenges in topical ocular drug delivery and to develop products that mimic tear film composition, ocular nanosystems (ONSs) with diameters ranging from 50 to 300 nm are being investigated as potential drug delivery technologies [14–15]. However, our formulation is not a drug delivery system (DDS) since it lacks pharmacological agents. Instead, it is a functional nanoemulsion designed to mimic the tear film’s properties and provide antioxidant and osmoprotective benefits, aiming to reduce DED symptoms. ONSs include nanoparticles such as nanoemulsions, liposomes, nanomicelles, and dendrimers, which can serve as carriers for both lipophilic and hydrophilic drugs. This allows for smaller doses and more precise drug targeting [16]. Nanoemulsions (NEs) show promise in improving drug bioavailability after topical ocular instillation, offering several benefits such as reducing the administration frequency, optimizing pharmacokinetic parameters, protecting against enzymatic degradation, and enhancing the stability of bioactive molecules [14,17–19].

Currently, three ophthalmic products based on NEs, Restasis^®^ (Allergan), Lacrinmune^®^ (Bausch & Lomb), and Ikervis^®^ (Santen). have been approved by regulatory agencies such as the FDA and EMA and are commercially available for treating DED [1]. These products are formulated with synthetic polymers and contain cyclosporine as the active ingredient [1]. NEs are designed as droplets that can potentially replenish the lipid layer in DED patients, mimicking the trilayered structure of the tear film, consisting of lipid, aqueous, and mucin layers [13,15,18,20]. According to the literature, the tear film compromises the stability of NE components. When NE is instilled, the oil nanodroplets merge with the lipid layer, the water in the formulation interacts with the aqueous layer of the tear film, and the surfactant interacts with the mucin layer [21–23].

The literature cites various lipids as components of the oily phase in nanoformulations of eye drops for treating DED [12,20–21,24–27]. These include mineral oil (found in Systane^®^ Complete eye drops) [28], castor oil, phospholipids (phosphatidylcholine and/or hydrogenated phospholipids), polyunsaturated fatty acids (PFAs), and medium-chain triglycerides. Among these, PFAs such as the omega–3 fatty acid alpha-linolenic acid, the omega–6 fatty acid linoleic acid, and the omega–9 fatty acid oleic acid are recognized for their potent antioxidant and anti-inflammatory properties [29–30]. These fatty acids are predominantly found in linseed oil (*Linum usitatissimum*, LO), making it a promising ingredient for NE formulations [31–33]. Clinical studies have demonstrated that fatty acids derived from linseed oil are effective in alleviating DED, primarily by modulating inflammatory responses and downregulating key molecular markers, such as interleukin-1 beta, matrix metalloproteinase-9, intercellular adhesion molecule 1, and tumor necrosis factor [34–36]. Downie et al. reported strong clinical evidence supporting the efficacy of nanolipid carrier eye drops infused with omega–3 fatty acids in stabilizing the tear film in patients with meibomian gland dysfunction [31].

In addition to PFAs, phospholipids are notable for their structure, which includes a hydrophilic part (phosphate groups) and a hydrophobic part (fatty acid chains). This unique configuration allows phospholipids to interact at the interface between the lipid and aqueous layers of the tear film, increasing its thickness and improving its stability [1,37]. This leads to immediate relief for patients with DED [28,38]. Recently, promising clinical results have been observed using liposomal spray (Tears Again^®^) for DED treatment [39]. This product primarily contains phosphatidylcholine, making it an effective therapeutic option for this ocular condition.

Artificial tear products commonly used to alleviate symptoms of DED contain various ingredients that promote osmoprotective and antioxidant effects. In this context, this study proposes the development of an ophthalmic nanoemulsion with multifunctional action, formulated from linseed oil (rich in PFAs) and lecithin (rich in phosphatidylcholine). The focus is on developing a functional ophthalmic nanoemulsion with antioxidant and osmoprotective effects by optimizing physical properties and stability for potential use in DED treatment.

## Experimental

### Materials and reagents

Linseed oil (code 430021), egg lecithin or egg ʟ-α-phosphatidylcholine (surfactant, ~60% TLC, code 61755), Kolliphor^®^ HS15 (co-surfactant, code 42966), benzalkonium chloride (cationic detergent, code B4136), disodium EDTA (C_10_H_14_N_2_Na_2_O_8_, code 114), DPPH (2,2-diphenyl-1-picrylhydrazyl, code D9132), triton-X100 (code T8787), phosphate buffered saline (PBS, code P4417), and F.A.M.E. Mix (C_8_–C_24_ unsaturated, code 18918) were purchased from Merck^®^/Sigma-Aldrich^®^ (Brazil). Hydrochloric acid (HCl, code R0101811000), sodium chloride (NaCl, code C1003460500), ethyl acetate (CH_3_COOCH_2_CH_3_, code R2800491000), and ethanol (C_2_H_5_OH, code R0401701000) were purchased from CRQ Produtos Químicos. Disodium phosphate (Na_2_HPO_4_·2H_2_O, code P.10.0513.012.00.27), petroleum ether (code P.10.0450.000.04.81), sodium dodecyl sulfate (C_12_H_25_SO_4_Na, code P.10.0645.000.00.27) were purchased from Dinâmica Química Contemporânea LTDA (Brazil). Potassium hydroxide (KOH, code HP09874RA), trypan blue (code AT06398SO), and methanol (CH_3_OH, code AM07445RA) were purchased from Êxodo Científica^®^ LTDA (Brazil). Monosodium phosphate (NaH_2_PO_4_, code 01414), phenolphthalein (C_20_H_14_O_4_, code 317415), and citric acid (C_6_H_8_O_7_·H_2_O, code 19228) were obtained from Neon LTDA (Brazil), Nuclear LTDA (Brazil), and AlphaQuimica LTDA (Brazil), respectively. Ascorbic acid (C_6_H_8_O_6_, product batch 032121-CF) was purchased from CMS Impex LTDA (Brazil). Thiazolyl blue tetrazolium bromide (MTT, code 976360) was purchased from Ludwig Biotec (Brazil). All analytical grade reagents were used as received from the supplier without further purification.

Ultrapure water was obtained from a Milli-Q^®^ (Merck Millipore) direct water purification system (18.2 MΩ·cm) and used for all aqueous solutions. L-929 cells (code 0188) were obtained from the Banco de Células do Rio de Janeiro (BCRJ)/ATCC (Brazil). RPMI Medium 1640 (code 31800022), fetal bovine serum (FBS, code 12657029), GlutaMAX (code 35050061), and penicillin–streptomycin (code 15140122) were purchased from GIBCO^TM^ (Brazil).

### Methodological basis: lecithin structure and emulsification process

Droplet size, homogeneity, and stability in colloidal systems are influenced by component proportions (surfactant, oil, and co-surfactant), emulsification methods (low-energy or high-energy techniques) [40–42], and intrinsic properties like linseed oil viscosity and lecithin’s molecular structure [43]. These last factors are crucial for organizing amphiphilic molecules and influencing the uniformity of micellar dispersions in ophthalmic nanoformulations.

Lecithin is a mixture of phospholipids and consists mainly of phosphatidylcholine, which typically forms liposomes (concentric lipid bilayers) rather than micelles (single-layered lipid structures) [43]. The hydrophilic portion of lecithin consists of phospholipids, while the presence of unsaturated and/or saturated fatty acids determines its hydrophobic characteristics, thereby influencing its hydrophilic–lipophilic balance (HLB) values [44]. However, understanding the HLB value requires clarifying the arrangement of the hydrophilic portions of the surfactant molecules [43]. Previous research suggests that the behavior of lecithin in solutions is directly influenced by the proportion of phospholipids and fatty acids in its composition, as well as the type of fatty acids, whether they are saturated or unsaturated [45–46]. Lecithins rich in unsaturated fatty acids (especially those with cis-double bonds, which create molecular kinks) tend to form micellar or disordered colloidal structures because these conformational kinks hinder tight molecular packing, thus destabilizing bilayer formation. Conversely, lecithins with a higher proportion of saturated phospholipids are more likely to organize into stable bilayer vesicles like liposomes. These structural features of lecithin play a crucial role in determining the physicochemical properties of the resulting formulations, including their thermodynamic stability, size distribution, and optical clarity [47–48].

The packing parameter concept (PPC) provides a theoretical framework for predicting the geometry of amphiphilic systems, based on the ratio of the surfactant molecule’s total volume, the area of its polar portions, and the length of its hydrophobic chain [49]. PPC values below 0.5 indicate the formation of monolayer micelles, whereas values between 0.5 and 1.0 favor liposome formation [49–50]. A value of *p* ≥ 0.5 has been reported for phosphatidylcholine, indicating that lecithin can form micelles and liposomes [51]. Considering the approximately 60% phosphatidylcholine content in the egg lecithin utilized in this study, its dissolution in the aqueous phase was prioritized due to the predominance of its hydrophilic (polar) properties. However, mixed micellar systems (comprising lipid mono- and bilayers) form due to the diverse array of fatty acids in lecithin, the positioning and unsaturation of which are not provided by the manufacturer. Therefore, experimental tests on lecithin concentration are necessary to achieve a monolayer, nanometric, uniform, and stable micellar structural arrangement. Rupp et al. reported the successful translation of the theoretical approach (by PPC) into a practical methodology by investigating variations in surfactant concentration during the emulsification process, which was also carried out in our study [43].

Liposomes are widely studied in ophthalmology for treating anterior and posterior eye diseases, including dry eye, keratitis, transplant rejection, uveitis, endophthalmitis, and proliferative vitreoretinopathy [52]. They can encapsulate hydrophilic and lipophilic drugs, enhancing bioavailability and targeted delivery [53]. However, their clinical use can be limited by stability issues like drug leakage, lipid oxidation, and vesicle aggregation, which depend on drug properties and bilayer composition [54]. These challenges are significant regarding chronic topical use. Conversely, micellar nanoemulsions, such as those in this study, offer better stability, smaller droplet size, and improved solubilization of hydrophobic compounds, making them more suitable for superficial ocular conditions like dry eye, where sustained bioavailability and tear film compatibility are vital [53]. Liposomes may also face practical issues due to interactions between the drug and phospholipids, affecting stability [52,54].

Moreover, heating linseed oil to the formulation temperature of 75 °C offers specific advantages, such as enhanced phase interaction and improved solubilization potential for hydrophobic drugs, without compromising its chemical integrity or bioactivity. No signs of oxidation or thermal degradation were observed at this temperature. TGA and DSC analyses confirm that degradation processes begin only above 340 °C, ensuring that the oil’s quality and functional properties are preserved during formulation. The optimal lecithin concentration was experimentally determined using a dilution method combined with a low-energy technique, ensuring the formation of uniform micelles ideal for ophthalmic applications, as detailed below.

### Preparation of ophthalmic nanoformulations

To obtain nanoemulsions for ocular application, three progressive studies were conducted to determine (1) the appropriate amounts of surfactant in the pre-emulsions, (2) the nanoformulation prepared in an ophthalmic vehicle (OV), and (3) its stabilization with a co-surfactant. The formulations prepared in the OV were sterilized using a polyethersulfone (PES) membrane filter (0.22 μm pore size) and stored at room temperature. The hydrophilic nature of PES facilitates filtration of lipid-based nanoemulsions, while potential retention of larger or deformable droplets may occur without compromising overall stability.

#### Surfactant concentration in the pre-formulations

Pre-nanoemulsions (25 mL) were prepared by varying the egg lecithin concentration from 1% to 5% (w/w) in 8% (w/w) oil in water, resulting in pre-emulsions with five distinct concentrations (described in Table S1, Supporting Information File 1). The oil and aqueous phases were heated separately to 75 °C. The mass of egg lecithin was initially dissolved in the aqueous phase at the concentration specified for each experiment. Once the surfactant was fully solubilized, preheated linseed oil (75 °C) was gradually added, and the mixture was homogenized using an Ultra Turrax (IKA T18, Dispersion element S18N-19G) at 10,000 rpm for 5 min. Then, the system underwent further homogenization with a Q125 Sonicator (QSonica, USA), using the 4435 probe (QSonica, USA) for 15 min (three cycles of 5 min each), at 100% amplitude, with pulses of 10 s on and 2 s off.

#### Ophthalmic nanoformulation

The OV was initially prepared by dissolving 560 mg of monosodium phosphate, 284 mg of disodium phosphate, 500 mg of sodium chloride, 100 mg of disodium EDTA, and 0.1 mg of benzalkonium chloride in 100 mL of Milli-Q water (pH 7.3). To formulate 10 mL of ophthalmic nanoemulsion (OphtNE), 3.75 mL of the optimized pre-formulation (after 24 h at rest) was diluted with 6.25 mL of OV, resulting in a nanoemulsion with a 3.70% (w/w) final concentration of LO. This mixture was homogenized using a Q125 Sonicator (QSonica, USA) with a 4435 probe (QSonica, USA) for 10 min (two cycles of 5 min each), set at 100% amplitude with pulses of 10 s on and 2 s off. This formulation, designated as OphtNE-3.70%, was obtained by diluting the optimized pre-emulsion in the OV, resulting in a final concentration of 3.70% (w/w) with 2.6% (w/v) LO (equivalent to 26 mg·mL^−1^). The detailed mass composition of OphtNE-3.70% is provided in Table S2 (Supporting Information File 1).

#### Stabilization of ophthalmic nanoformulation with co-surfactant

The stabilization of the surfactant was enhanced by incorporating Kolliphor® HS15 as a co-surfactant at a concentration of 1% (w/v), as described in the study by Dukovski and colleagues [21]. For the preparation of a 10 mL ophthalmic nanoemulsion containing the co-surfactant (with a final concentration of 3.66% w/w), 3.75 mL of optimized pre-formulation (after 24 h of rest) was diluted in 6.25 mL of the OV, which had 100 mg of Kolliphor^®^ HS15 pre-solubilized through magnetic stirring. The resulting formulation was then homogenized using a Q125 Sonicator (QSonica, USA), with a 4435 probe (QSonica, USA) for 10 min (two cycles of 5 min), set to 100% amplitude with 10 s on and 2 s off pulses. This nanoemulsion, designated as OphtNE-3.66%(K1%), was obtained by diluting the optimized pre-emulsion in the OV to a final concentration of 3.66% (w/w), containing 2.6% (w/v) LO (equivalent to 26 mg·mL^−1^ or 26,000 µg·mL^−1^) and added with 1% (w/v) Kolliphor® HS15 (indicated by the “K”). The detailed mass composition of OphtNE-3.66%(K1%) is provided in Table S2 (Supporting Information File 1).

### Characterization techniques for linseed oil, pre-formulation, and ophthalmic nanoformulations

Linseed oil (LO), pre-formulations, and ophthalmic nanoformulations (OphtNE-3.70% and OphtNE-3.66%(K1%)) were characterized using the following techniques to assess their chemical, physical, biological (in vitro), and morphological properties. These characterizations were performed to ensure the quality and stability of the formulations and to evaluate their suitability for ophthalmic applications.

#### Gas chromatograph with flame ionization detection (GC-FID)

LO’s fatty acid methyl esters were transesterified as described in [37–38]. Briefly, 1 g of LO was mixed with 100 mL methanolic KOH (2.5% w/v) and heated at 70 °C under magnetic stirring (3,000 rpm) for 1 h. Afterward, 100 mL of ethyl acetate was added, and the mixture was stirred at 3,000 rpm for 5 min. The resulting mixture was transferred to a separation funnel to remove the denser phase. The supernatant was washed three times with 10% (w/v) citric acid and then concentrated via rotary evaporation. A 2 µL aliquot of the supernatant was injected in split injection mode (1:10 in ethyl acetate) into a gas chromatograph with flame ionization detection (GC-FID) model 7290 A (Agilent Technologies, USA). The column used was a SP^®^-2560 capillary GC column (100 m × 0.25 mm × 0.20 µm) (Supelco). The injector temperature was maintained at 230 °C, with helium (He) serving as the carrier gas at a flow rate of 1 mL·min^−1^. The oven temperature was programmed to increase from 150 to 300 °C at a rate of 5 K·min^−1^. Peak identification was performed using the F.A.M.E Mix analytical standard (C_4_–C_24_ unsaturated), and chromatogram analysis was carried out using OpenLab software (Agilent Technologies, USA). The fatty acid (FA) composition of linseed oil (LO) is summarized below in Table 1.

#### Viscosity and density

The viscosity and density of LO were measured using an SVM 3000/G2 kinematic viscometer (Anton Paar, USA). The measurements were performed at two temperatures: 25 °C (by ASTM D7042-21 standard) and 75 °C, with each measurement repeated in triplicate to determine the mean ± standard deviation.

#### Acid value for linseed oil

The acid value (AV) of LO was determined following the American Oil Chemists Society (AOCS) Cd 3d-63 method. For quantification, 5 g of LO was dissolved in 50 mL of a petroleum ether and ethanol mixture (1:1 v/v ratio) at 25 °C under magnetic stirring. The sample was titrated with a standardized ethanolic KOH solution (*f* = 1.07), using phenolphthalein (1%, w/v) as the indicator. The AV of the LO was calculated using Equation 1, with all determinations conducted in triplicate to obtain the mean ± standard deviation:


[1] $$\text{acid value [mg}\cdot {\text{g}}^{-1}\text{]}=\frac{\left(V_{\text{A}}-V_{\text{B}}\right)\cdot \left[\text{KOH}\right]\cdot 56.1\text{ g}\cdot {\text{mol}}^{-1}}{M_{\text{A}}},$$

where *V*_A_ is the volume of KOH solution required for the titration of the LO sample (mL), V_B_ is the volume of KOH solution required for blank solvent titration (mL), [KOH] is the concentration of ethanolic KOH solution (0.093 mol·L^−1^), *M*_A_ is the mass of the LO sample used (g), and 56.1 g·mol^−1^ is the molecular weight of KOH.

#### Thermal analysis

Thermogravimetric (TG) and differential scanning calorimetry (DSC) analyses of LO (20.5 mg) were performed using a Simultaneous Thermal Analyzer (STA) 449 F3 Jupiter (NETZSCH, Germany). The samples were scanned from 25 to 700 °C at a heating rate of 10 K·min^−1^ under a nitrogen (N_2_) atmosphere with a flow rate of 50 mL·min^−1^, using sealed aluminum pans (T181206 and T181128).

#### Droplet size, polydispersity index, zeta potential, and conductivity

Droplet size and polydispersity index (PdI) of the formulations were measured 24 h after preparation using dynamic light scattering with a Zetasizer Nano-ZS ZEN 3600 (Malvern Instruments, UK) at 25 °C. Before analysis, the samples were diluted 1:1000 with Milli-Q water, a standard dilution used to prevent multiple scattering without compromising micelle integrity. Measurements were performed at a 173° backscatter detection angle. Zeta potential and electrical conductivity were determined by electrophoretic mobility using the same instrument at 25 °C. Calculations were based on the Smoluchowski model. All measurements were performed in triplicate and expressed as mean ± standard deviation.

#### pH Value

The pH value of the pre-formulation and nanoformulations was measured using a digital pH meter (HI2221, Hanna Instruments, BR) equipped with a calibrated glass electrode and a temperature sensor. The electrode and sensor were immersed in the samples, and pH readings were recorded once the measurements stabilized.

#### Transmission electron microscopy

The morphology of the nanoemulsions was analyzed using transmission electron microscopy (TEM) with a MORGAGNI 268D (FEI Company, USA), operated at 80 kV. Before imaging, the samples were sonicated in an ultrasound bath for 15 min, and a drop of the suspension was placed onto a copper grid (200 mesh) coated with formvar/carbon. Excess liquid was removed with filter paper, and the samples were counterstained with 2.5% uranyl acetate (w/v). The grids were then dried under vacuum for 24 h before TEM analysis.

#### Long-term stability test

The long-term stability of the nanoformulations was assessed by storing the samples in hermetically sealed glass vials at 25 °C, protected from light and kept at rest. The average droplet diameter (size-weighted), polydispersity index, and zeta potential were measured on days 1, 30, 45, and 60 after preparation, following the methodologies previously described. All measurements were performed in triplicate to calculate the mean ± standard deviation.

#### Antioxidant activity

The antioxidant activity of the linseed oil-based nanoformulation was assessed using the 2,2-diphenyl-1-picrylhydrazyl (DPPH) free radical photocolorimetry method [55–56]. A 100 µL aliquot of DPPH solution (0.3 mmol·L^−1^ in 99% ethanol) was mixed with 100 µL of ophthalmic nanoformulation, corresponding to linseed oil concentrations of 0.65 to 10.4 mg·mL^−1^ (corresponding to 25, 50, 100, 200, 300, and 400 µL of nanoformulation per mL of ethanol). The reaction mixture was incubated in a 96-well plate at 25 °C for 1 h, protected from light. Absorbance was then measured at 518 nm using a Multiskan Go UV spectrophotometer (Thermo Fisher Scientific, FI). Ascorbic acid (3% w/v, prepared in the ophthalmic vehicle) was used as the positive control. The experiment was performed in quadruplicate, and antioxidant activity (DPPH radical inhibition percentage) was calculated using Equation 2 [57]. The IC_50_ (the concentration required to achieve 50% inhibition) of the radical scavenging activity was determined via linear regression analysis [58].


[2] $$\text{DPPH inhibition }[\%\text{]}=\frac{{\text{Abs}}_{\text{control}}-\left({\text{Abs}}_{\text{sample}}-{\text{Abs}}_{\text{blank}}\right)}{{\text{Abs}}_{\text{control}}}\cdot 100,$$

where Abs_control_, represents the absorbance of the DPPH radicals, Abs_sample,_ represents the absorbance of the nanoformulation, and Abs_blank_ represents the absorbance of the sample without DPPH (sample + ethanol).

#### Cytotoxicity assay

L929 fibroblast cells were cultured in RPMI 1640 medium supplemented with 10% (v/v) fetal bovine serum (FBS), 1% (v/v) penicillin–streptomycin, and 1% (v/v) GlutaMAX, maintained under standard conditions (5% CO_2_ at 37 °C). Cell viability was determined using the trypan blue exclusion method. The in vitro cytotoxicity of the sterile nanoformulations was evaluated according to the guidelines of the International Organization for Standardization (ISO 10993-5:2009) [59], using the MTT assay to assess cell viability based on metabolic activity.

L929 fibroblasts were seeded in 96-well plates at 2 × 10^4^ cells per well. After 24 h, the medium was replaced with 100 µL of fresh medium containing sterile ophthalmic nanoformulations, providing final linseed oil concentrations of 0.65 to 13 mg·mL^−1^ (25, 50, 100, 200, 300, 400, and 500 µL nanoformulation per mL of medium).

After incubation for 24 and 72 h, the treatments were removed, and 100 µL of MTT solution (0.5 mg·mL^−1^) was added to each well, followed by 3 h of incubation under standard culture conditions. To solubilize the blue-violet formazan crystals, 100 µL of 10% (w/v) sodium dodecyl sulfate solution, acidified with 0.1 mol·L^−1^ HCl, was added. After 24 h, the absorbance was measured at 570 nm using a Multiskan Go microplate reader (Thermo Fisher Scientific, FI). The positive control consisted of cells treated with 1% (v/v) Triton X, while the negative control comprised cells treated with only the supplemented RPMI medium.

The percentage of metabolically active cells was calculated by comparing the absorbance of the samples to that of the negative control. Linear regression analysis was used to determine the maximum non-cytotoxic concentration (MNCC70), defined as the concentration at which 70% cell viability was maintained. The assay was performed in triplicate across four independent experiments.

#### Graphs and statistical analysis

All data in tables and graphs are presented as mean ± standard deviation. Graphs were generated using OriginPro 2022 software (OriginLab Corporation, USA). The statistical analysis (StatSoft Inc., 2011, Tulsa, USA) was performed using analysis of variance (ANOVA), and mean differences were compared using the Tukey test. Statistical significance was set at *p* < 0.05.

For the cytotoxicity data analysis, technical replicates were collapsed using the median. Outliers were identified and excluded based on the interquartile range (IQR) method: values above Q3 + 1.5 × IQR or below Q1 − 1.5 × IQR were removed for each experimental group. Normality and homoscedasticity were assessed using the Shapiro–Wilk and Levene’s tests, respectively. A three-way ANOVA was then performed, considering the factors exposure time (24 and 72 h), concentration (0.65 to 13 mg·mL^−1^), and treatment (OphtNE-3.70% and OphtNE-3.66%(K1%)). Two-way interactions were further examined using post hoc tests, including one-way ANOVA, estimated marginal means, and Tukey’s honestly significant difference (HSD) test. When appropriate, the Bonferroni correction was applied for multiple comparisons. Statistical significance was defined as *p* < 0.05. Data processing and statistical analyses were performed using R v.4.5.1 [60], specifically the packages “tidyverse” v.2.0.0 [61] and “rstatix v.0.7.2 [62]. Supplementary statistical data are provided in spreadsheets and can be accessed in Supporting Information File 2.

## Results and Discussion

### Physicochemical properties of linseed oil

The identification and quantification of fatty acids (FAs) in linseed oil (LO) are presented in Table 1. Linolenic acid is the most abundant, constituting 46.7%, followed by oleic acid (17.7%), linoleic acid (14.5%), behenic acid (7.4%), erucic acid (5.6%), palmitic acid (5.0%), and stearic acid (3.3%). Heating LO to 75 °C did not significantly affect the relative quantities of these fatty acids. Unsaturated FAs (~79%) remained dominant over saturated FAs (~21%), consistent with the results reported by Qiu et al. [63] and Rahiminezhad and colleagues [64]. Among the FAs in LO, α-linolenic acid (ALA, omega–3), oleic acid (OA, omega–9), and linoleic acid (LA, omega–6) are recognized as essential bioactive compounds, making LO valuable for medicinal applications. Notably, ALA is metabolized into eicosapentaenoic acid (EPA, C20:5) and docosahexaenoic acid (DHA, C22:6), providing polyunsaturated fatty acids (PUFAs) that are vital for human health [65–66]. PUFAs are well-known for their therapeutic potential, particularly their anti-inflammatory and antioxidant properties [30], showing promising results in systemic and topical treatment [33,67–68], including for dry eye disease [35–36,69].

**Table 1:** Identification of fatty acid methyl esters, retention times, fatty acid profiles, carbon chain structures, and relative quantities in linseed oil (Sigma-Aldrich^®^).

Ester	Retention time (min)	Fatty acid	Carbon chain (carbon atoms:unsaturation)	Relative quantity (%)^a^

methyl palmitate	22.405	palmitic acid	16:0	4.9
methyl stearate	26.368	stearic acid	18:0	3.3
methyl oleate	27.581	oleic acid	18:1	17.7
methyl linoleate	29.336	linoleic acid	18:2	14.5
methyl linolenate	31.407	linolenic acid	18:3	46.7
methyl behenate	34.030	behenic acid	22:0	7.4
methyl erucate	35.271	eructic acid	22:1	5.6

^a^Determined from the relative peak area ([the peak area assigned to each fatty acid/the total peak areas of the fatty acids] × 100).

**Figure 1:**
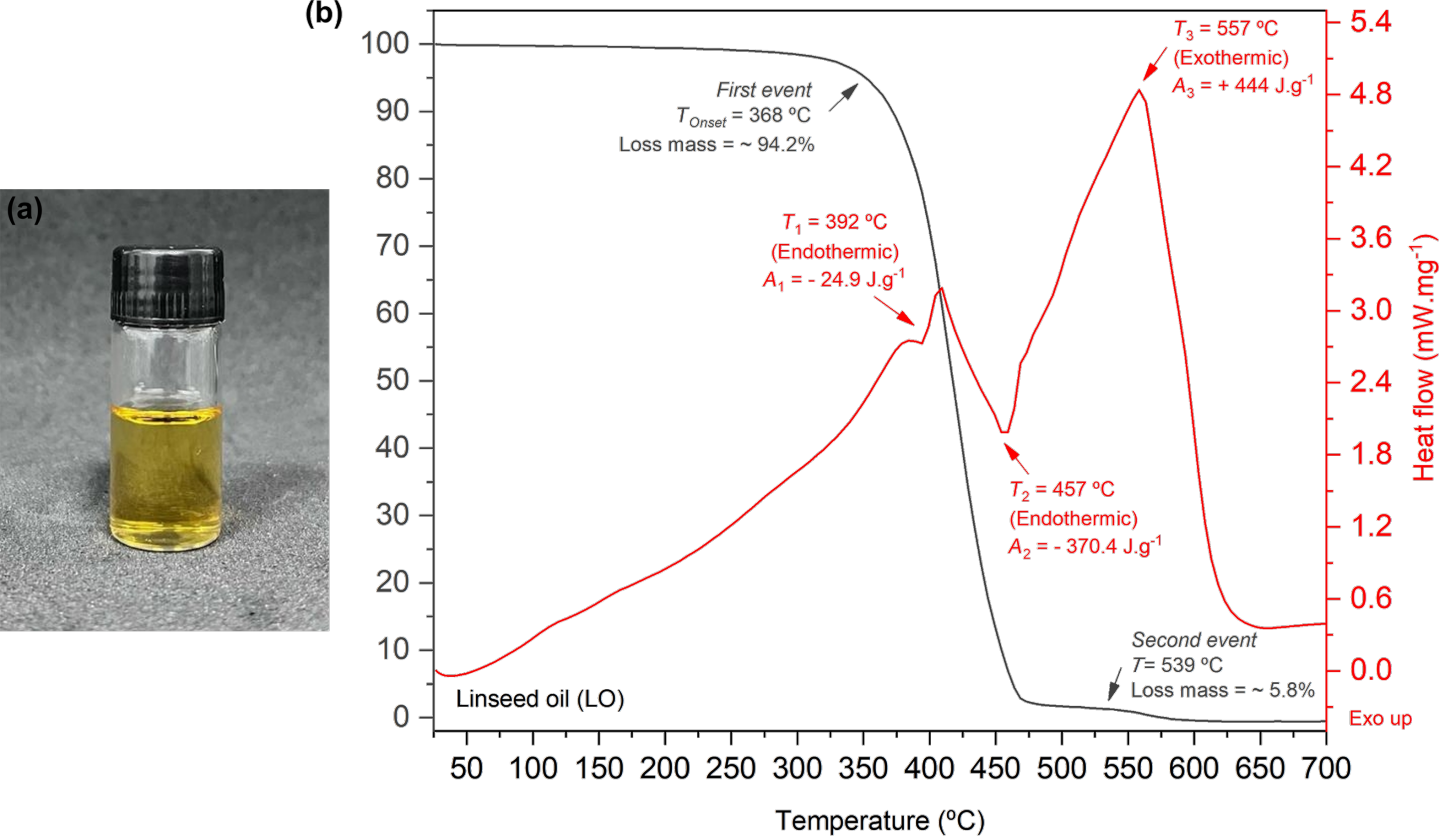
Linseed oil (Sigma-Aldrich^®^): (a) image of the oil after heating to 75 °C and (b) TGA/DSC curves.

The viscosity, density, and AV of LO at both room temperature (25 °C) and elevated temperature (75 °C) are summarized in Table 2. Visually, heating LO to 75 °C did not result in any noticeable changes in color or turbidity; the oil maintained its original yellowish hue and translucent appearance, as shown in Figure 1a.

**Table 2:** Density, viscosity, and acid value of linseed oil (Sigma-Aldrich^®^) at 25 and 75 °C.

	Temperature (°C)	Density (g·cm^−3^)	Dynamic viscosity (mPa·s^−1^)^a^	Acid value (mg·g^−1^)^a^

linseed oil (Sigma-Aldrich^®^)	25	0.93	40.76 ± 0.22^1^	1.74 ± 0.04^1^
	75	0.89	9.91 ± 0.01^2^	1.66 ± 0.07^1^

^a^Values are expressed as mean ± standard deviation (n = 3), and equal superscript numbers indicate no statistically significant difference (α = 0.05) by Tukey’s test.

The observed density of LO at 25 °C was consistent with the values reported by Rahiminezhad and colleagues [64]. However, increasing temperature reduced LO density (by ca. 4%). This reduction can be attributed to the increased molecular motion of the oil’s long fatty acid chains (saturated and unsaturated) when heated. The increased motion causes greater intermolecular spacing, leading to an increase in the oil’s volume. As a result, the oil’s mass-to-volume ratio (density) decreases, as observed for vegetable oils [70]. Raising the temperature of LO to 75 °C resulted in a statistically significant reduction (75.7%) in viscosity (*p* < 0.05). Linseed oil consists predominantly of unsaturated fatty acids, characterized by long hydrocarbon chains containing double bonds between carbon atoms. At higher temperatures, these double bonds relax and undergo configurational changes from cis to trans, leading to distortions in the molecular structure [71–72]. This results in weaker intermolecular interactions, reducing the oil’s viscosity [73]. A lower viscosity in LO can facilitate the formation and stability of micelles by allowing surfactant molecules to move more freely, promoting their rapid formation and maintaining stability within the emulsion [74].

An important quality parameter of LO is the free fatty acid (FFA) content, determined by the AV. A lower AV signifies a reduced FFA content, indicating higher oil quality and enhanced bioactive functionality, making it well-suited for medical applications. The AV measured for LO at 25 °C was significantly lower than the value reported by Tariq et al. [75], indicating that the LO used in this study was of higher quality and free from deterioration, oxidation, and adulteration. Heating the LO to 75 °C had no significant impact on the AV, which remained stable at this temperature. This finding aligns with the results obtained through GC-FID analysis, confirming that heating to 75 °C did not affect the quality of the oil. To provide a comprehensive understanding of the thermal behavior of LO, the TG and DSC curves are presented in Figure 1b. The TGA curve for the LO revealed two distinct thermal mass loss events. The first event occurs between 340 and 490 °C, where a significant mass reduction (~94.2%) is observed, with the degradation temperature marked at 368 °C (*T*_Onset_). This primary degradation phase involves lipolysis, the breakdown of triglycerides into fatty acids and glycerol. Following lipolysis, glycerol decomposes into acrolein, a compound known for its toxicity to humans, as well as the breakdown of unsaturated fatty acid chains. The second mass loss event (~5.8%) occurs between 539 and 600 °C and is attributed to the decomposition of saturated fatty acids and low-molecular-weight compounds generated during earlier thermal degradation processes.

The DSC curve for LO exhibited three distinct calorimetric events. The first endothermic peak (*T*_1_ = 392 °C) is likely attributed to the presence of polyunsaturated fatty acids, as confirmed by GC-FID analysis. As the temperature rises, the double bonds in these unsaturated fatty acids undergo relaxation and changes from cis to trans configuration, distorting the molecular structure. This leads to weaker intermolecular interactions, which is reflected in a reduction in oil viscosity (as shown in Table 2) and a decrease in the oil’s melting point. The second endothermic peak (*T*_2_ = 457 °C) is attributed to the rupture of double bonds in the unsaturated fatty acids, resulting in the formation of glycerol and shorter-chain fatty acids. Finally, the third exothermic peak (*T*_3_ = 557 °C) corresponds to the molecular rearrangement and decomposition of the oil into simpler compounds, such as aldehydes, ketones, and aromatic hydrocarbons.

These findings affirm the thermal stability of linseed oil at 75 °C, showing no significant changes in acid value or oxidation signs as per GC-FID analysis. TGA and DSC results reveal that degradation only occurs above 340 °C, ensuring that the formulation temperature does not affect the oil’s quality or bioactivity. Overall, the oil’s consistent thermal stability, fatty acid profile, and reduced viscosity under heat facilitate the formation of nanoemulsions, thereby enhancing its therapeutic benefits for DED, where antioxidant and anti-inflammatory effects are crucial.

### Structural organization of micelles in the ophthalmic nanoformulations

To establish the stability of nanoemulsions, it is essential to identify a region in the ternary diagram where the concentrations of oil, water, and surfactant/co-surfactant are ideal for the formation of nanometer-sized droplets, minimizing the risk of coalescence or phase separation. A viable strategy to streamline the extensive series of formulations and to locate the optimized region in the ternary diagram involves diluting an emulsion, if the component proportions in its formulation are adjusted to enhance stability. In this context, as an initial approach, different concentrations of lecithin were evaluated in pre-formulations (pre-emulsions). The results for particle size, polydispersity index, electrical conductivity, zeta potential, and pH for all formulations (pre-formulations and ophthalmic nanoformulations) obtained are summarized in Table 3. The particle intensity distribution profiles of the pre-formulations are presented in Figure S1 of Supporting Information File 1.

**Table 3:** Results of weighted average droplet diameter (nm), polydispersity index (PdI), electrical conductivity (µS·cm^−1^), zeta potential (mV), and pH for pre-formulations and ophthalmic nanoformulations (with and without co-surfactant).

Procedures for manufacturing nanoformulations	Formulation code^a^	[Total] (%, w/w)^b^	LO (%, w/v)	Weighted mean of sizes (nm)^c^ *_F_*_(5,18) = 15.24,_ *_p_*_ = 0.0023_	PdI	Conductivity (µS·cm^−1^)^c^ _F(5,18) = 74.04,_ *_p_*_ < 0.0001_	Zeta potential (mV)^c^ _F(5,18) = 67.34,_ *_p_*_ < 0.000001_	pH

pre-formulation (pre-emulsion)	O/W(L-1%)	8.2	8	470.2 ± 66.9^1^	0.389	9.0 ± 0.8^1^	−33.3 ± 0.8^1^	4.7
	O/W(L-2%)	9.1	8	347.6 ± 1.5^1,2^	0.383	9.3 ± 2.0^1^	−40.7 ± 0.7^2,3^	4.6
	O/W(L-3%)	9.9	8	308.0 ± 2.3^3^	0.252	11.7 ± 0.1^2^	−39.9 ± 0.2^2^	4.6
	O/W(L-4%)	10.7	8	227.6 ± 2.8^3,4^	0.238	14.8 ± 0.2^3^	−41.6 ± 0.8^2,3^	4.4
	O/W(L-5%)	11.5	8	222.3 ± 3.7^3,4^	0.244	14.6 ± 0.7^3^	−42.8 ± 1.5^2,3^	4.5
ophthalmic nanoformulation	OphtNE-3.70%	3.70	2.6	173.1 ± 3.9^5^	0.146	18.6 ± 0.7^4^	−44.7 ± 0.2^4^	6.9
ophthalmic nanoformulation with co-surfactant	OphtNE-3.66%(K1%)	3.66	2.6	70.8 ± 0.8^6^	0.126	17.3 ± 0.8^4^	−37.6 ± 0.8^2^	6.8

^a^Lecithin is represented by L and Kolliphor^®^ HS15 by K; ^b^[Total] = [(mass of LO + mass of L)/mass of water + mass of LO+ mass of L] × 100; ^c^values are mean ± SD; equal superscript numbers (1–6) show no significant difference (α = 0.05) per Tukey’s test.

The successful sterile filtration of nanoemulsions through 0.22 μm PES membranes demonstrates the viability of this method for ophthalmic use. PES membranes are hydrophilic, have low lipid affinity, reduce fouling, and are compatible with aqueous nanoemulsions. Although droplet size was below the pore size, larger or aggregated droplets might be retained due to deformability, causing minor phase loss or changes. Despite an initial droplet size larger than 220 nm, the surfactant-rich interface enables passage through the membrane. The filtration also helps exclude larger, unstable droplets, improving size uniformity. PES membranes are thus effective for sterilizing nanoemulsions without affecting stability or function [76–78].

The droplets in the pre-formulations exhibited a weighted average diameter ranging from 470 nm (lower surfactant content) to 222 nm (higher surfactant content), demonstrating the influence of lecithin concentration on the droplets’ hydrodynamic diameter (*F* = 67.34, *p* < 0.000001, one-way ANOVA). Initially, when the surfactant content was 1% and 2% (w/w) in the 8% (w/w) pre-formulation, the droplet hydrodynamic diameter exceeded 300 nm, showing a multimodal distribution of intensities with PdI values above 0.3. This suggests that the lecithin content in the O/W(L-1%) and O/W(L-2%) pre-formulations is insufficient to effectively reduce surface tension, resulting in incomplete droplet coverage and subsequent coalescence. As the lecithin concentration in the pre-formulations was increased, the hydrodynamic diameter of the droplets and PdI values decreased, indicating improved uniformity. PdI values below 0.3 suggest greater size homogeneity [79]. Under the same ultrasound emulsification conditions, the droplet diameter reaches an optimal point, indicating surfactant saturation at the droplet interface. This reduces surface tension by fully covering the interfacial surface area, thereby stabilizing the emulsion structure [80–81]. Among the pre-formulations, O/W(L-3%) demonstrated optimal performance, with a hydrodynamic diameter of approximately 300 nm and a PdI value of 0.252. This monomodal intensity distribution confirmed efficient surfactant coverage. Further lecithin additions, such as in O/W(L-4%) and O/W(L-5%), led to free micelle formation due to surfactant excess, resulting in smaller droplets but multimodal distributions. Similar results were reported in previous studies that reported the influence of surfactant concentration on the formation and stability of nanoemulsions [82–84].

In summary, variations in lecithin concentration assessed in pre-formulations containing linseed oil delineate distinct profiles concerning droplet diameter distribution, delineating three discernible phases. The initial phase (represented by pre-formulations O/W(L-1%) and O/W(L-2%)) exhibits inadequate lecithin levels to entirely coat the oil droplet interfaces. The subsequent phase (represented by pre-formulation O/W(L-3%)) reveals a saturation point of lecithin concentration at the oil droplet interfaces, ensuring optimal surfactant utilization. Finally, the third phase (illustrated by pre-formulations O/W(L-4%) and O/W(L-5%)) manifests an overabundance of lecithin, resulting in the formation of micellar structures within the pre-formulations.

As electrical conductivity is a physicochemical parameter modulated by surfactant concentration in emulsified systems [85]. The measured conductivity values corroborate earlier findings, with statistical significance confirmed by one-way ANOVA (*F* = 74.04, *p* = 0.000026), as shown in Table 3. In the initial phase, the electrical conductivity of pre-formulations O/W(L-1%) and O/W(L-2%) exhibited no significant changes with increasing lecithin concentration. Moving into the second phase, the O/W(L-3%) pre-formulation demonstrated a notable 21% increase in dispersion conductivity, indicating statistical significance compared to formulations with lower lecithin concentrations. Transitioning to the third phase, characterized by excess lecithin, both O/W(L-4%) and O/W(L-5%) pre-formulations exhibited a statistically significant 21% rise in electrical conductivity compared to O/W(L-3%). This elevation is attributed to free micelles capable of facilitating electron displacement within the aqueous dispersion. The enhanced conductivity facilitated by electron transport species can foster chemical interactions with contacting materials (such as primary packaging) or expedite degradation processes, particularly given the acidic nature of the pre-formulations.

Variations in zeta potential (−33.3 to −44.7 mV) reflect the influence of surface-active functional groups, particularly the phosphate groups in lecithin. This effect was statistically significant (*F* = 15.24, *p* = 0.00235, one-way ANOVA), as reported in Table 3. These groups impart a negative charge to the droplet surfaces, attributed to the amphiphilic nature of lecithin, whose polar head contains the phosphate anion ([PO_4_]^3-^). Müller [86] suggested that when the absolute value of the zeta potential exceeded 30 mV (in module), the droplets in the system would be stabilized by strong electrostatic repulsion. All pre-formulations exhibited zeta potential values exceeding −30 mV, as shown in Table 3.

The lecithin concentration significantly influenced the negative charge density on the droplets’ surface. This effect was evident in the pre-formulation containing 2% (w/w) lecithin (O/W (L-2%)), where the surfactant coating begins to form on the droplet interface. While cationic and anionic nanoemulsions are commonly explored in drug delivery systems due to their distinct electrostatic properties, the present formulation was not designed as a drug delivery vehicle. Instead, it functions as a bioactive nanoemulsion intended to stabilize the tear film and provide antioxidant and osmoprotective effects. Cationic emulsions are known to enhance residence time on the ocular surface via electrostatic interactions with negatively charged mucins [25,87–88], while anionic nanoemulsions offer better compatibility with the components of tear fluid [2,89]. Based on this, an anionic profile was selected in this study to optimize retention and interaction with the ocular environment, aiming to support tear film function and alleviate dry eye symptoms through the multifunctional properties of LO. Although the physicochemical profile is compatible with nanosystems often used in drug delivery, no pharmacological agent was incorporated in this study.

All pre-formulations displayed a pH below 5, indicating their acidic nature. This acidity can be attributed mainly to LO, which has a pH of 4.1 and is the major component of pre-formulations at an 8% (w/w) concentration. The increase in lecithin concentration did not significantly impact the pH of the pre-formulations. While pH and ionic strength directly affect the electrical charge of system components [83,90], the observed zeta potential values were consistently related to the surfactant amount surrounding the droplets.

Several criteria were considered to determine the optimal lecithin concentration for forming coarse pre-emulsions: (1) achieving a mean hydrodynamic droplet diameter close to 300 nm to ensure the efficacy and safety of ocular administration [91–92]; (2) ensuring droplet uniformity with a monodisperse profile [93]; (3) maximizing lecithin’s performance in forming LO droplets through monolayer micelles [81]; (4) avoiding excess surfactant molecules to maintain biocompatibility [94–95]; and (5) maintaining a negative surface charge density greater than −30 mV for stability dispersion [84]. Based on these criteria, the O/W(L-3%) pre-formulation was identified as the most suitable candidate for further investigations in the development of ophthalmic nanoformulations for DED.

After optimizing the pre-formulation parameters, a simple dilution process of O/W(L-3%) combined with ultrasonic emulsification resulted in a reduction of the hydrodynamic diameter of the droplets by approximately 44%, leading to a decrease in the PdI values to 0.146 (see Table 3), thereby achieving a narrower monomodal distribution profile, a characteristic feature of nanoemulsions obtained through ultrasound [96].

The dilution process of the O/W(L-3%) system facilitated a more uniform distribution of lecithin molecules around the surface of the LO droplets, effectively reducing the interfacial tension. This process, coupled with low-energy ultrasound, allowed for the reduction of the droplet diameter [97]. Furthermore, diluting the pre-formulation increases the average distance between the droplets, thereby decreasing electrostatic repulsion interactions between them [83]. As a result, the OphtNE-3.70% nanoformulation produces smaller and more stable droplets, attributed to the reduced interfacial tension and the reorganization of lecithin molecules, which minimize aggregation over time.

However, a high density of ionic surfactants at the system interface requires careful consideration. Strong electrostatic repulsion within the dispersion can generate dipole moments between droplets, increasing surfactant mobility on their surfaces [98]. This mobility may create gaps at the interface, ultimately promoting coalescence and causing destabilization over time. To avoid this effect, Kolliphor^®^ HS15, a non-ionic hydrophilic surfactant, was added as a second surfactant. The addition of this co-surfactant aimed to stabilize and slightly reduce the negative charge density in the droplet surface [99]. Furthermore, the combination of lecithin with another hydrophilic surfactant has a greater capacity to form micelles [43]. Kolliphor was selected due to its common use in ophthalmic products and FDA approval for concentrations up to 5% [88]. This co-surfactant reduced the zeta potential by approximately 16% compared to OphtNE-3.70%. Despite this reduction, OphtNE-3.66%(K1%) maintained zeta potential values below −30 mV, ensuring dispersion stability and supporting its use in ophthalmic nanoformulations.

After diluting the O/W(L-3%) pre-formulation in VO, a significant reduction of approximately 75% in droplet size was observed following the addition of Kolliphor^®^ HS15. This substantial size reduction can be attributed to the surfactant’s chemical structure, which effectively anchors onto the surfaces of the oil droplets. Additionally, the ultrasound process further contributes to size reduction by promoting droplet fragmentation. The smaller droplet size increases the surface-area-to-volume ratio, enhancing electrostatic shielding. This effect is particularly pronounced when the non-ionic co-surfactant occupies the droplet surface. These findings are consistent with Dukovski et al., who also highlighted the role of Kolliphor^®^ HS15 in reducing droplet size in nanoemulsions [21].

**Figure 2:**
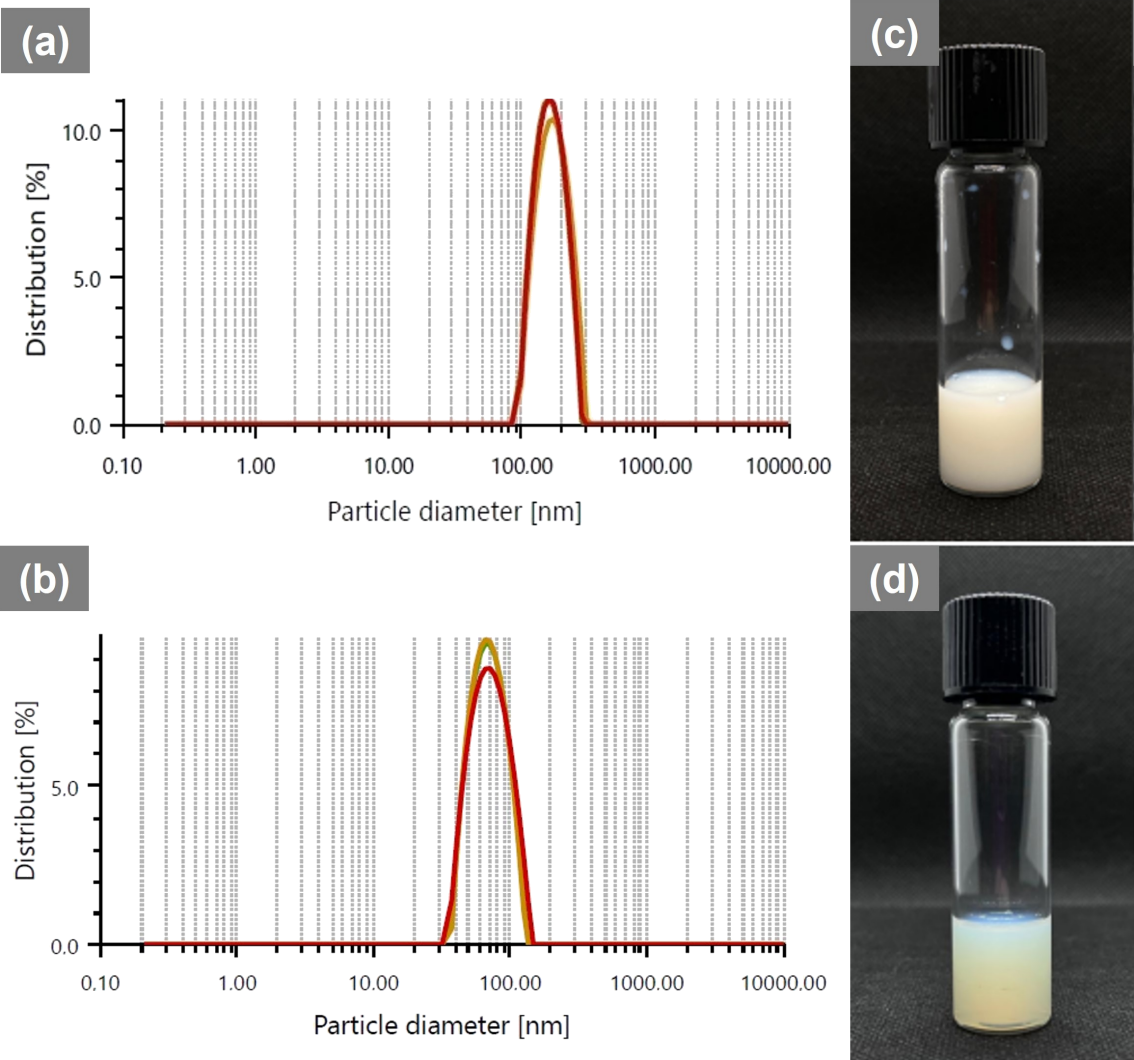
Droplet diameter distribution graphs and macroscopic appearance after 24 h at rest of sterile ophthalmic nanoformulations: (a, c) OphtNE-3.70%, and (b, d) OphtNE-3.66%(K1%).

The sterile nanoformulations OphtNE-3.70% and OphtNE-3.66%(K1%) exhibited droplet sizes ranging from larger than 50 nm to smaller than 300 nm, which are considered suitable for permeating the ocular barriers, indicating their potential as multifunctional systems for hydrophobic drug delivery to the eye [14,100]. Figure 2a and Figure 2b display the droplet diameter distribution plots for OphtNE-3.70% and OphtNE-3.66%(K1%), respectively.

The electrical conductivity of OphtNE-3.70% and OphtNE-3.66%(K1%) was higher than that of the O/W(L-3%) pre-formulation due to using VO as a diluent. VO contains electrolytes, particularly NaCl, which improves conductivity. In contrast, diluting the ophthalmic formulations in pure distilled water may cause variations in conductivity, which can compromise the consistency and effectiveness of your applications [101]. When preparing ophthalmic nanoformulations, VO guarantees more stable and reproducible conductivity conditions, with values consistently below 50 μS·cm^−1^ [102–103], compared to literature-reported nanoemulsions for DED treatment based on castor oil and 1,2-dimyristoyl-*sn*-glycerol-3-phosphocholine with a conductivity of 68.9 μS·cm^−1^ [20]. Another study described the development of a nanoemulsion-based gel containing moxifloxacin hydrochloride to treat conjunctivitis with a conductivity of 390 μS·cm^−1^ [104]. In comparison to both studies, the ophthalmic nanoformulations developed demonstrated lower conductivity, indicating good tolerance and no ocular irritation.

Preparing the OphtNE-3.70% and OphtNE-3.66%(K1%) nanoformulations involved diluting the O/W(L-3%) pre-formulation in OV. This process increased the pH of the nanoformulations by buffering salts such as NaHPO_4_ and Na_2_PO_4_. Maintaining the pH within the range of 6.5–8.0 ensures ocular compatibility, avoiding irritation and enhancing drug absorption [92,105–106].

### Morphological, stability, and biological properties (in vitro) of ophthalmic nanoformulations

The pre-formulation emulsion (O/W systems with 1–5% lecithin) appeared homogeneous and milky white, typical of stable oil-in-water systems with fine droplets (Figure S2 in Supporting Information File 1). In contrast, the sterile ophthalmic nanoformulations OphtNE-3.70% and OphtNE-3.66%(K1%) (Figure 2c and Figure 2d, respectively) also exhibited a milky appearance but with distinct optical clarity, influenced by their smaller and more uniform nanodroplet size distributions. These visual attributes support the colloidal stability and successful transition from pre-formulation to nanoemulsion via ultrasonic processing.

The OphtNE-3.70% nanoformulation (containing only lecithin) forms a monomolecular film at the oil–water interface. When light interacts with this finely dispersed droplet system, it scatters in multiple directions, giving the nanoemulsion its characteristic milky opaque appearance. Similar optical properties have been documented in the literature for lecithin-based nanoemulsions with droplet sizes of approximately 150 nm [21,107]. The OphtNE-3.66%(K1%) nanoformulation appeared more translucent than the OphtNE-3.70% nanoformulation. This difference can be attributed to two main factors: (1) the significant reduction in droplet size and (2) the improved uniformity of the nanodroplets. These factors minimize light scattering, resulting in a more transparent nanoemulsion.

**Figure 3:**
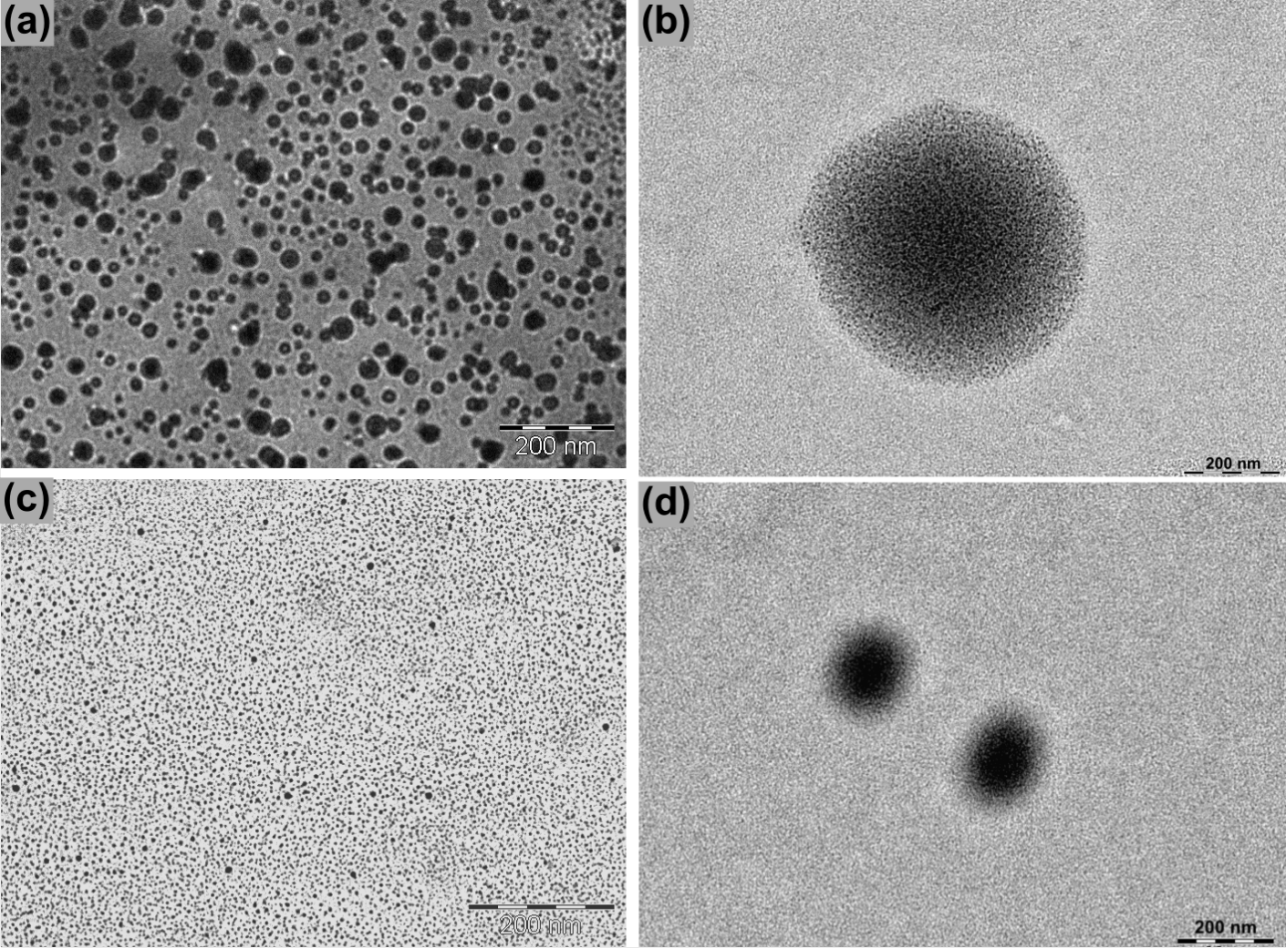
TEM micrographs of ophthalmic nanoformulations: OphtNE-3.70% (a, b) and OphtNE-3.66%(K1%) (c, d) at low (a, c) and high (b, d) magnification, showing nanodroplet and micellar morphology.

The macroscopic difference between the ophthalmic nanoformulations OphtNE-3.70% and OphtNE-3.66%(K1%) is further supported by the TEM micrographs presented in Figure 3a and Figure 3c, respectively. These micrographs reveal spherical droplets in both formulations, but distinct differences in size and dispersion uniformity are evident. OphtNE-3.70% displays larger and more heterogeneous particles, whereas OphtNE-3.66%(K1%) exhibits smaller, uniformly distributed droplets. This observation aligns with the PdI results shown in Table 3, confirming that the addition of Kolliphor^®^ HS15 improved both homogeneity and droplet size reduction. This feature is crucial for ensuring the stability of formulations and achieving uniformity in the dosage unit, a key requirement for ophthalmic applications [27]. The high-magnification images in Figure 3b and Figure 3d reveal the micellar structure, characterized by dense cores resulting from the presence of linseed oil and less dense peripheries formed by the interfacial layer, which consists of the surfactant and co-surfactant. This structural organization underscores the effective interaction between the formulation’s components.

**Figure 4:**
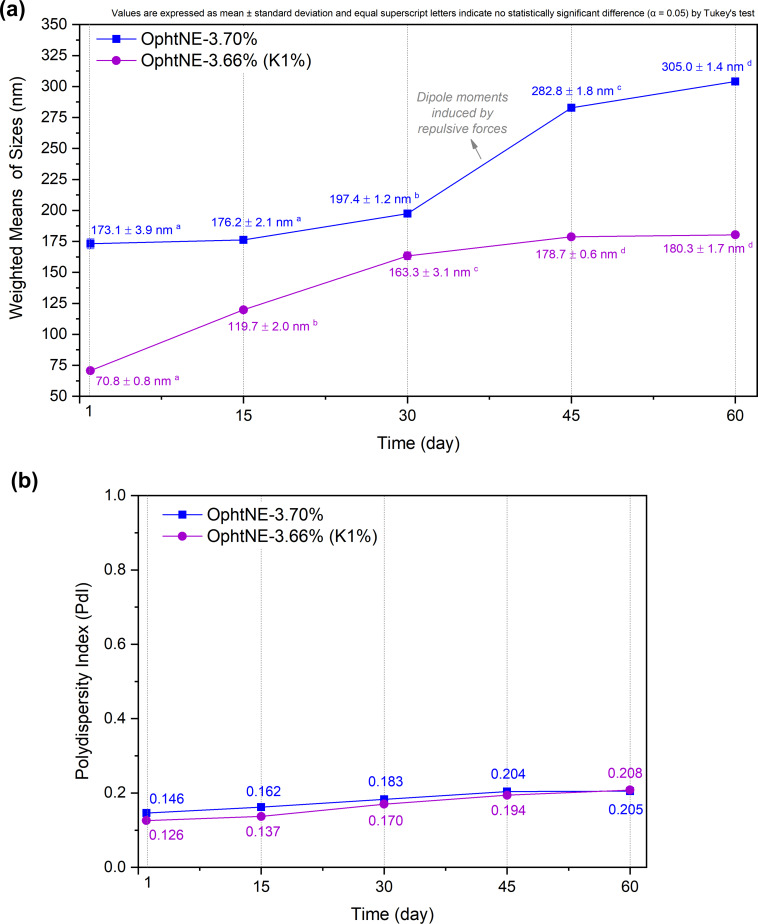
Changes in (a) average droplet size and (b) polydispersity index of OphtNE-3.70% and OphtNE-3.66%(K1%) nanoformulations over 60 days at room temperature. Data are shown as means ± standard deviation (*n* = 3) with statistical significance (*p* < 0.05).

To ensure the prolonged stability of the OphtNE-3.70% and OphtNE-3.66%(K1%) nanoformulations, droplet size monitoring was carried out fortnightly for 60 days. Figure 4a and Figure 4b present the weighted means of particle size and PdI, respectively, during storage.

Emphasis should be placed on the phenomenon known as Ostwald ripening, commonly observed in colloidal systems, particularly in nanoemulsions [108–109]. During Ostwald ripening, smaller particles, which have a higher surface-area-to-volume ratio, diffuse and aggregate into larger droplets, leading to coalescence and an overall increase in droplet size. This dynamic process leads to a tendency for particle enlargement over time, as observed in both ophthalmic nanoformulations. However, this phenomenon was more pronounced in the first 15 days of OphtNE-3.66%(K1%) due to its smaller droplet size than OphtNE-3.70%.

For OphtNE-3.70%, relative stability was observed up to approximately 30 days of storage. After this period, a significant increase in the droplet size occurred, showing a 76% increase compared to the initial size, as depicted in the graph. This phenomenon can be attributed to the formation of induced dipole moments between negatively charged droplets of lecithin, creating repulsive forces that, over time, cause the droplets to coalesce due to the formation of interfacial gaps on their surfaces. However, the addition of Kolliphor^®^ HS15 in OphtNE-3.66%(K1%) mitigated this effect by redistributing surface charges, improving interfacial organization. As a result, the formulation exhibited more consistent droplet size behavior over time, with only moderate increases in size and PdI. Although some variation occurred, no phase separation or macroscopic instability was observed, indicating acceptable colloidal stability under storage conditions. The PdI values of the ophthalmic nanoformulations remained relatively stable throughout the storage period, staying close to 0.2, indicating a uniform particle size distribution. This controlled droplet size profile in nanoemulsions can be attributed to the simple dilution process from pre-formulations. As reported in the literature, the storage of diluted nanoemulsions after treatment in a microfluidizer exhibited significantly greater stability compared to undiluted nanoemulsions [41].

**Figure 5:**
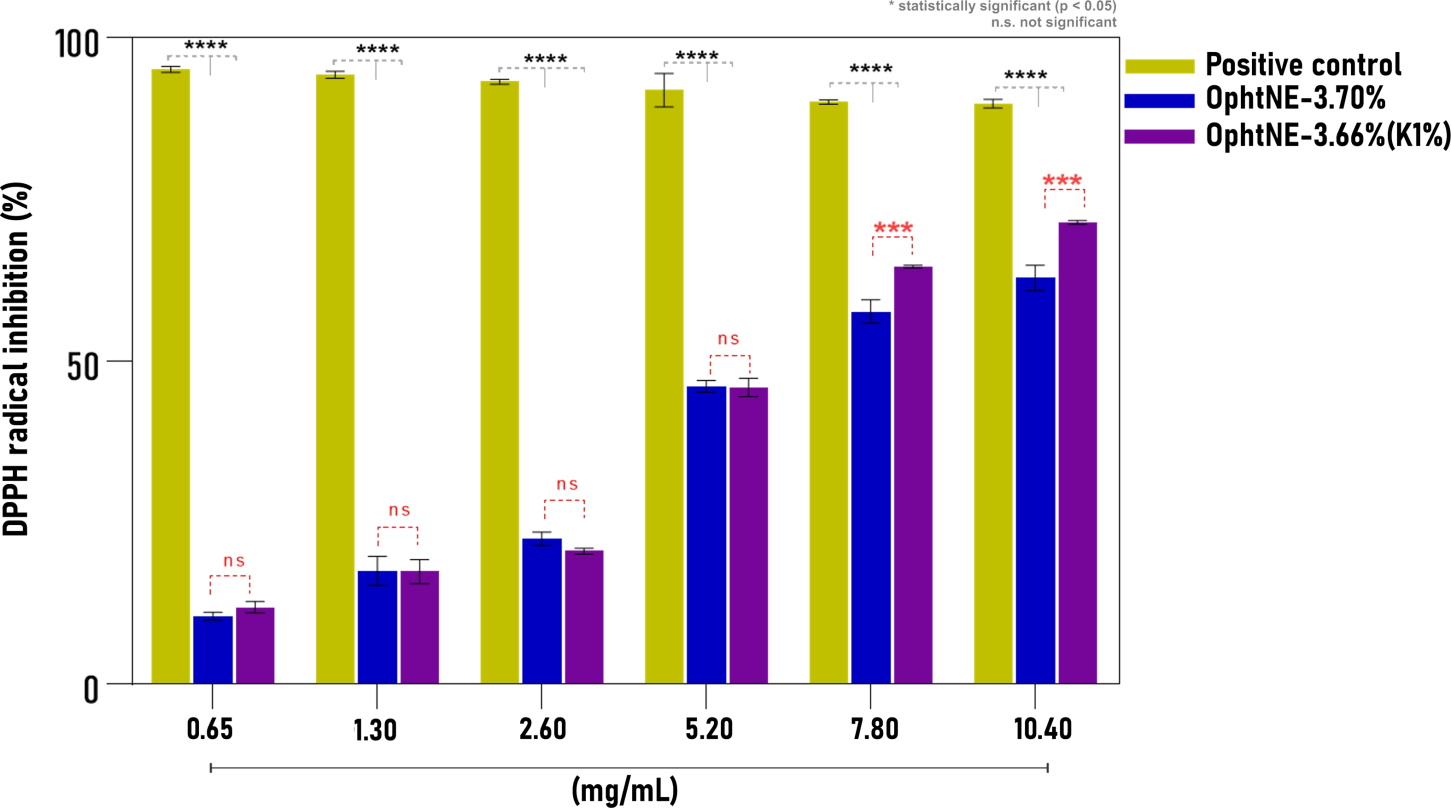
Antioxidant activity (DPPH assay) of OphtNE-3.70% and OphtNE-3.66%(K1%) at different concentrations (0.65–10.4 mg·mL^−1^), with ascorbic acid as the positive control. Data are mean ± SD. *p < 0.05; n.s., not significant.

The antioxidant potential of ophthalmic nanoformulations was evaluated using the DPPH assay, a well-established method for assessing the free radical-scavenging capacity of bioactive substances through a visible color change in the reaction medium. Typically, the greater a compound’s ability to reduce DPPH radicals, the higher its antioxidant effect [55]. The antioxidant activity of OphtNE-3.70% and OphtNE-3.66%(K1%) nanoformulations was evaluated through these tests, as shown in Figure 5. The results demonstrated a statistically significant dose-dependent response. The antioxidant activity observed is attributed to the presence of linseed oil at 3% (w/v) in the formulations, rich in α-linolenic acid and other bioactive components. As the concentration of nanoformulations increased, their antioxidant activity increased, compared to ascorbic acid (positive control). The essential omega–3 fatty acid is known for its ability to neutralize free radicals, which contributes to protection against cellular oxidative stress by synthesizing EPA and DHA [28,65,110]. Supporting these findings, a multicenter, double-blind, randomized clinical study conducted by Downie et al. developed a nanoemulsion-based tear formulation containing linseed oil and trehalose [31]. This nanoformulation provided additional ocular surface protection, highlighting its potential as a promising product for DED.

When comparing the nanoformulations, a statistically significant difference in antioxidant activity was observed beginning at an LO concentration of 7.80 mg·mL^−1^, with OphtNE-3.66%(K1%) showing greater antioxidative potential due to its smaller nanodroplet size. The relationship between droplet size and antioxidant capacity has been highlighted in previous studies, where emulsified systems exhibited superior antioxidant performance compared to non-emulsified essential oils [55]. Additionally, antioxidant nanoemulsions containing α-tocopherol have been reported as effective strategies for glaucoma treatment [111].

The DPPH 50% inhibition activity (IC_50_) was estimated at 9.56 mg·mL^−1^ for OphtNE-3.70% and 9.16 mg·mL^−1^ for OphtNE-3.66%(K1%). The linear regression equations used to determine the IC_50_ values for the antioxidant activity of ophthalmic nanoformulations are provided in Figure S3 in Supporting Information File 1. These antioxidant activities highlight the potential of OphtNE-3.70% and OphtNE-3.66%(K1%) to protect cells from oxidative damage caused by free radicals. This protection is essential for preserving cellular integrity and functionality, ensuring accurate and reliable results in cytotoxicity experiments. Notably, at LO concentrations lower than 7.80 mg·mL^−1^, no statistical difference in antioxidant performance was observed between the two formulations, emphasizing their similar efficacy in cellular protection.

Given that the data satisfied the assumptions of normality and homoscedasticity, a three-way ANOVA was conducted to assess L929 cell viability based on absorbance measurements, which showed significant effects of concentration and time, as well as the interactions treatment:concentration and concentration:time (Table 4). Cell viability was influenced by the synergistic effects of nanoformulation concentration and exposure time. Increased absorbance values indicated cell proliferation, whereas decreased values signified cell death.

**Table 4:** Three-way ANOVA of the effects of treatment, concentration, and time, and their interactions, on MTT assay absorbance in L929 cells.^a^

Effect	DF_n_	DF_d_	*F*	*p*	*p* < 0.05	ges

treatment (OphtNE-3.70% and OphtNE-3.66%(K1%))	1	99	82.887	9.83 10^−15^	*	0.456
concentration (mg·mL^−1^)	8	99	60.025	1.39 10^−34^	*	0.829
time (h)	1	99	0.107	0.744	ns	0.001
treatment:concentration	8	99	8.734	5.81 10^−9^	*	0.414
treatment:time	1	99	1.595	0.21	ns	0.016
concentration:time	8	99	2.924	0.006	*	0.191
treatment:concentration:time	8	99	0.797	0.607	ns	0.060

^a^*: significant (*p* <0.05); ns: not significant.

The two-way ANOVA applied to the treatment:concentration interaction was statistically significant at all analyzed levels (Table S3 in Supporting Information File 1), supporting the trends observed in the graphical representation. The results of the one-way ANOVA for the significance levels are presented in Table S4 in Supporting Information File 1.

**Figure 6:**
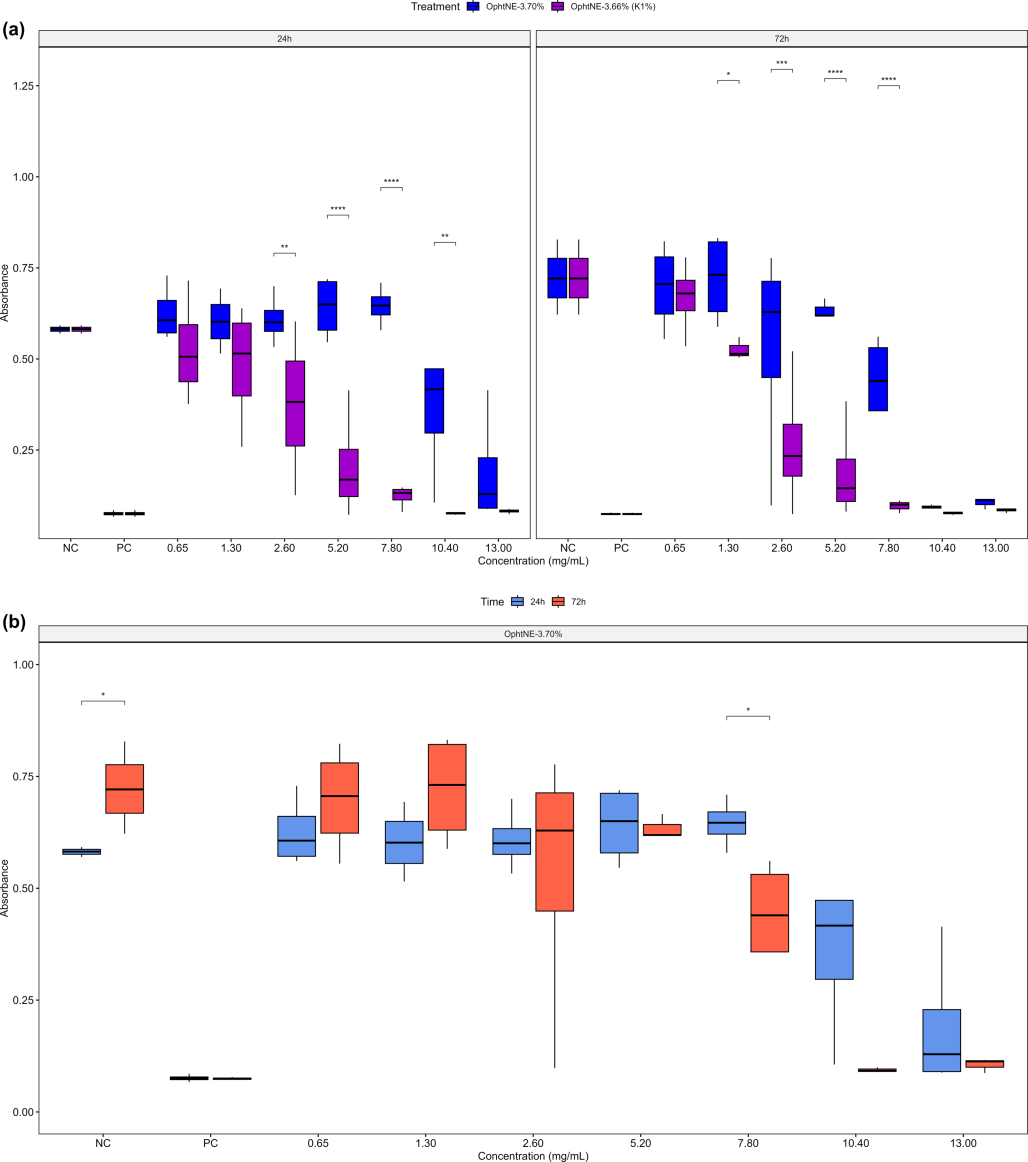
MTT assay absorbance for L929 cells: (a) boxplots for OphtNE-3.70% and OphtNE-3.66%(K1%) by treatment:concentration interaction at 24 and 72 h; (b) boxplots for OphtNE-3.70% by concentration:time interaction. *p < 0.05; n.s., not significant.

The boxplot in Figure 6a displays a characteristic dose-dependent response pattern, where increasing concentrations of the nanoformulations lead to a progressive decrease in absorbance values and, consequently, in cell viability, observed at both 24 and 72 h of exposure. This behavior is indicative of a cytotoxic effect, likely associated with the accumulation of nanoformulation components in the extracellular environment.

OphtNE-3.70% maintained high cell viability, comparable to the negative control, up to a concentration of 7.80 mg·mL^−1^ (300 µL·mL^−1^), suggesting good tolerability after 24 h of exposure. This observation aligns with previous reports indicating that lecithin-containing formulations are generally well tolerated by non-tumor cells, due to their structural similarity to plasma membrane phospholipids [42,112]. Such compatibility may facilitate spontaneous fusion with the membrane or passive endocytosis without triggering inflammatory or apoptotic responses. However, at concentrations of 10.40 mg·mL^−1^ and above, a marked reduction in absorbance values and, consequently, in cell viability was observed. This effect may be primarily attributed to: (i) the relatively high sample volume applied to the wells (400 µL·mL^−1^), potentially limiting oxygen and nutrient diffusion; and (ii) lipid overload, which could disrupt local osmotic balance and alter membrane fluidity and integrity, thereby promoting necrosis or delayed apoptosis [113–114].

From a biochemical perspective, this high lipid load may enhance lipid peroxidation and disrupt membrane lipid domain organization, affecting associated proteins and the cytoskeleton, thereby further compromising cell viability. These effects become particularly evident at higher concentrations, such as 13 mg·mL^−1^, where absorbance values approach those observed in the positive control (non-viable cells). Under this condition, the high proportion of formulation relative to the RPMI medium (500 µL of formulation per well) amplifies the physicochemical stress on the cells, impairing the maintenance of homeostasis and promoting irreversible loss of membrane integrity.

In contrast, OphtNE-3.66%(K1%), which contains Kolliphor^®^ HS15 as a co-surfactant, exhibited more pronounced cytotoxicity even at lower concentrations. Cell viability was comparable to the negative control only at 1.30 mg·mL^−1^, decreasing sharply at higher concentrations. This effect may be attributed to the smaller size of the nanodroplets, which increases the specific surface area, thereby enhancing interactions with the cell membrane and facilitating particle internalization via endocytosis [115]. Previous studies have linked colloidal nanosystems to increased mitochondrial instability and the activation of apoptotic pathways, even in normal cells [116–117].

Regarding the concentration:time interaction, the two-way ANOVA revealed statistical significance only for OphtNE-3.70% (Table S5 in Supporting Information File 1). This effect was further examined through a one-way ANOVA, pooling exposure times to assess the influence of concentration, which confirmed the significance of this factor (Table S6 in Supporting Information File 1). This finding suggests that, for this nanoformulation, exposure time exerts a more pronounced influence on the cellular response compared with the OphtNE-3.66%(K1%) (containing Kolliphor^®^ HS15). The boxplot in Figure 6b illustrates an upward trend in absorbance values over time, particularly at lower concentrations, which may indicate either cellular recovery following initial exposure or adaptation to the external stimulus. This pattern was also observed in the negative control, supporting the experimental consistency. Notably, at LO concentrations of 7.80 and 10.40 mg·mL^−1^, a reduction in absorbance value and, consequently, in L929 cell viability was detected at 72 h but not at 24 h. This observation indicates a delayed cytotoxic effect, potentially associated with intracellular accumulation of nanoemulsion components, mitochondrial dysfunction, or oxidative stress induced by prolonged exposure [118]. Collectively, these findings underscore the importance of considering multiple parameters in the development of ophthalmic nanoformulations, including lipid concentration, application volume, exposure time, and formulation composition. Fine-tuning these variables is essential to ensure therapeutic efficacy without compromising cellular safety. Furthermore, the relevance of long-term in vivo studies is highlighted as a means to predict potential adverse events that may arise from repeated topical clinical applications.

Cell viability results above 70% are generally considered acceptable by the ISO 10993-5:2009. [59]. Table 5 summarizes the maximum non-cytotoxic concentration (MNCC) for L929 cells exposed to the developed ophthalmic nanoformulations. A comparative analysis of the results revealed that only the OphtNE-3.66%(K1%) nanoformulation showed decreased tolerance, despite a slight increase in its MNCC with prolonged exposure.

**Table 5:** Results for 70% maximum non-cytotoxic concentration (MNCC_70_) of nanoformulations OphtNE-3.70% and OphtNE-3.66%(K1%) after 24 and 72 h of culture.

Ophthalmic nanoformulations	Maximum Non-Cytotoxic Concentration (MNCC)			

	24 h (short exposure)		72 h (long exposure)	

	MNCC_70_ (mg·mL^−1^)	MNCC_70_ (µL·mL^−1^)	MNCC_70_ (mg·mL^−1^)	MNCC_70_ (µL·mL^−1^)

OphtNE-3.70%	7.80	300	7.80	300
OphtNE-3.66%(K1%)	1.30	50	2.60	100

Cell-based assays are widely accepted alternatives to Draize tests (in vivo), given the evidence of the correlation between cytotoxicity and ocular damage, particularly ocular irritation [119]. The scientific community strongly advocates integrating in vitro tests to generate robust data, developing alternative methods, and reducing reliance on animal testing [120]. The biocompatibility and safety of linseed oil have been consistently demonstrated in biomedical applications, particularly in ophthalmology [121–123]. In a randomized clinical trial involving patients with dry eye disease, a nanoemulsion containing linseed oil was associated with a significantly lower incidence of treatment-related adverse events compared to a conventional artificial tear formulation [121]. These findings support the therapeutic potential of linseed oil-based formulations in the management of ocular surface disorders.

Beyond its lubricating properties, linseed oil has also been investigated as a vehicle for ocular drug delivery, showing the capacity to prolong pre-corneal residence time and enhance local bioavailability [122]. Although specific concentrations of linseed oil within nanoemulsions are rarely disclosed, efficacy is largely attributed to nanotechnology-driven production of ultrafine, stable oil droplets.

Oral supplementation with linseed oil capsules (1–2 g/day) has further demonstrated clinical benefits in patients with Sjögren’s syndrome, reducing ocular surface inflammation and alleviating disease-related symptoms [123]. In addition, a novel artificial tear nanoemulsion enriched with linseed oil produced significant improvements in ocular staining parameters, including corneal and conjunctival staining scores [123]. Preclinical evidence also supports these findings. In a rabbit model, a linseed oil-containing formulation exerted a robust inhibitory effect on experimentally induced ocular inflammation, reinforcing both its biocompatibility and therapeutic value [122].

However, correlating the concentration of nanoformulations with their actual application volume is essential, particularly for topical applications such as eye drops. Considering that the average volume of an ophthalmic drop is approximately 50 µL [9,11,27], the resulting concentration of active components administered per dose would be around 1.30 mg·mL^−1^. Notably, cytotoxic effects in L929 cells were observed only at concentrations exceeding this limit, suggesting satisfactory in vitro biocompatibility at the intended dose. Furthermore, both nanoformulations contain 2.6% (w/v) linseed oil, a bioactive lipid rich in polyunsaturated fatty acids with recognized anti-inflammatory and antioxidant activities. This concentration ensures sufficient delivery of the oil’s functional components while maintaining physicochemical and biological stability. Therefore, the observed multifunctional behavior (antioxidant capacity and cytocompatibility) can be attributed, at least in part, to the presence of linseed oil at this optimized level, reinforcing its therapeutic potential in the DED treatment.

The nanoformulations have several benefits: (i) antioxidant activity shown by DPPH assays, crucial for reducing oxidative stress in DED; (ii) high compatibility with fibroblasts, indicating safety for the eye; (iii) nanoscale size with low polydispersity, improving penetration and stability; (iv) negative zeta potential, promoting stability and interaction with mucins; and (v) a pH value close to that of natural tears, ensuring tolerability. These features make them promising carriers for treating DED.

## Conclusion

This study presents a scalable pre-formulation strategy based on lecithin-stabilized pre-emulsions refined by tip ultrasound. This approach enabled the development of two sterile ophthalmic nanoformulations, namely, OphtNE-3.70% (lecithin only) and OphtNE-3.66%(K1%) (lecithin with Kolliphor^®^ HS15). Both nanoemulsions demonstrated antioxidant activity and cytocompatibility, supporting their potential use in the DED treatment.

Linseed oil was characterized by GC-FID, acid value, viscosity, and thermal analyses (TGA/DSC), confirming its identity, purity, and thermal stability (even after mild heating), thus validating its suitability for nanoemulsion preparation. This pre-formulation ensures effective emulsification and regulation of droplet size, thereby supporting multifunctional properties (such as antioxidant and cytocompatible responses), while also facilitating sterile and scalable manufacturing suitable for ophthalmic drug products. Both nanoformulations were developed with 2.6% (w/v) (equivalent to 26 mg·mL^−1^) of the linseed oil, a bioactive lipid known for its antioxidant and anti-inflammatory effects. This optimized concentration ensures therapeutic relevance within the typical ocular drop volume (~50 µL), while maintaining cytocompatibility. The multifunctional behavior observed in vitro is thus closely associated with the preserved activity of the oil in its nanoformulated form.

Although this study focused on formulation design and in vitro characterization, future work should include comparative biological assays between the native and nanoformulated oil to better elucidate their respective contributions. Two methodological limitations were also noted: osmolality was not assessed, and in vitro inflammatory markers were not analyzed. These aspects should be addressed in future studies to confirm ocular tolerability and to explore the anti-inflammatory potential of the nanoemulsions, particularly considering the bioactivity of linseed oil fatty acids.

## Supporting Information

Supporting Information File 1 contains a detailed table of the mass composition of the pre-formulations (O/W(L-1%), O/W(L-2%), O/W(L-3%), O/W(L-4%), and O/W(L-5%)) and ophthalmic nanoformulations (OphtNE-3.70% and OphtNE-3.66%(K1%)), along with visual and experimental data supporting the article. Included are macroscopic images of the pre-formulations, particle size distribution profiles, and linear regression curves used to determine antioxidant activity (IC_50_). Also, this file contains the two-way and one-way ANOVA tables for the statistically significant treatment:concentration and concentration:time interactions identified in the three-way ANOVA, based on MTT assay absorbance data from L929 cells. Supporting Information File 2 contains the data processing workflow and statistical analyses, including the Shapiro–Wilk test for assessing normality, outlier removal, and Levene’s test for evaluating homoscedasticity.

File 1 Supplementary tables and figures.

File 2 Complementary statistical data.

## Data Availability

All data that supports the findings of this study is available in the published article and/or the supporting information of this article.
