# Supplementary material for: Multifunctional anionic nanoemulsion with linseed oil and lecithin: a preliminary approach for dry eye disease
Source: Beilstein J Nanotechnol. 2025 Oct 2;16:1711–33. doi: 10.3762/bjnano.16.120 (PMC12498254; doi:10.3762/bjnano.16.120)
Supplement: File 1 — Supplementary tables and figures. [file Beilstein_J_Nanotechnol-16-1711-s001.pdf]

BEILSTEIN JOURNAL OF NANOTECHNOLOGY

Supporting Information

for

Multifunctional anionic nanoemulsion with linseed oil and lecithin: a preliminary approach for dry eye disease

Niédja Fittipaldi Vasconcelos, Almerinda Agrelli, Rayane Cristine Santos da Silva, Carina Lucena Mendes-Marques, Isabel Renata de Souza Arruda, Priscilla Stela Santana de Oliveira, Mércia Liane de Oliveira and Giovanna Machado

Beilstein J. Nanotechnol. **2025,** *16,* 1711–1733. doi:10.3762/bjnano.16.120

Supplementary tables and figures

License and Terms: This is a supporting information file under the terms of the Creative Commons Attribution License (https://creativecommons.org/licenses/by/4.0). Please note that the reuse, redistribution and reproduction in particular requires that the author(s) and source are credited and that individual graphics may be subject to special legal provisions.
The license is subject to the *Beilstein Journal of Nanotechnology* terms and conditions: (https://www.beilstein-journals.org/bjnano/terms)

SUPPLEMENTARY TABLES AND FIGURES

Table S1: A detailed description of the mass composition of the pre-formulation emulsions (8% w/w LO in water).

Pre-Formulation Emulsions Code*	Composition (in grams)			[Total] (%, w/w)***
	Milli-Q water	Linseed Oil**	Egg lecithin	
O/W(L-1%)	25	2	0.25	8.2
O/W(L-2%)	25	2	0.5	9.1
O/W(L-3%)	25	2	0.75	9.9
O/W(L-4%)	25	2	1.0	10.7
O/W(L-5%)	25	2	1.25	11.5

* Lecithin is represented by the letter L

** Density of oil at 75 ºC = 0.89 g.cm-1

*** [Total] = [(Mass of Linseed Oil + Mass of Egg lecithin) / Mass of Water + Mass of Linseed Oil + Mass of Egg lecithin] x 100

Table S2: Mass composition of the ophthalmic nanoformulations prepared with 2.6% (w/v) linseed oil (equivalent to 26 mg·mL^{-1} or 26,000 µg·mL^{-1}) in the ophthalmic vehicle.

Ophthalmic Nanoformulations	Composition					Total mass (g)	[Total] (%, w/w)***
	Ophthalmic Vehicle (g)	Kolliphor® HS15 (g)	Milli-Q water (g)	O/W(L-3%)** (g)			
				Linseed Oil	Egg lecithin		
OphtNE-3.70%	6.25	-	3.38	0.26	0.11	10.0	3.70
OphtNE-3.66%(K1%)*	6.25	0.1	3.38	0.26	0.11	10.1	3.66

* Kolliphor® HS15, indicated by K

** Density of the mixture = 0.995 g.cm-1

*** [Total] = [(Mass of Linseed Oil + Mass of Egg lecithin) / Mass of Water + Mass of Linseed Oil + Mass of Egg lecithin + Mass of Ophthalmic Vehicle] x 100

Figure S1: Droplet diameter distribution graph for pre-formulation emulsions: (A) O/W(L-1%), (B) O/W(L-2%), (C) O/W(L-3%), (D) O/W(L-4%), and (E) O/W(L-5%).

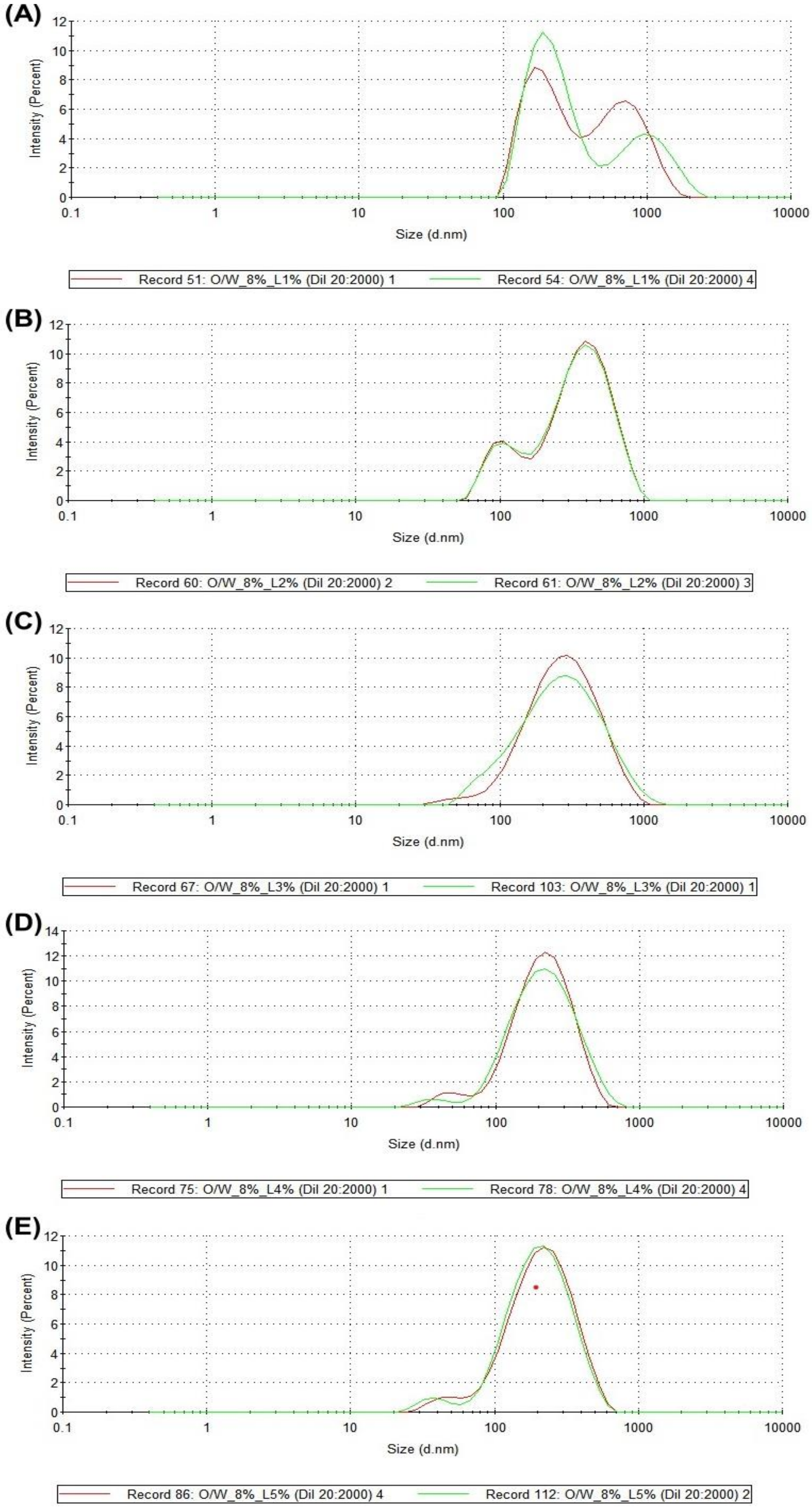

Figure S2: The macroscopic appearance of pre-formulation emulsions after 24 h reveals a homogeneous, milky-white appearance.

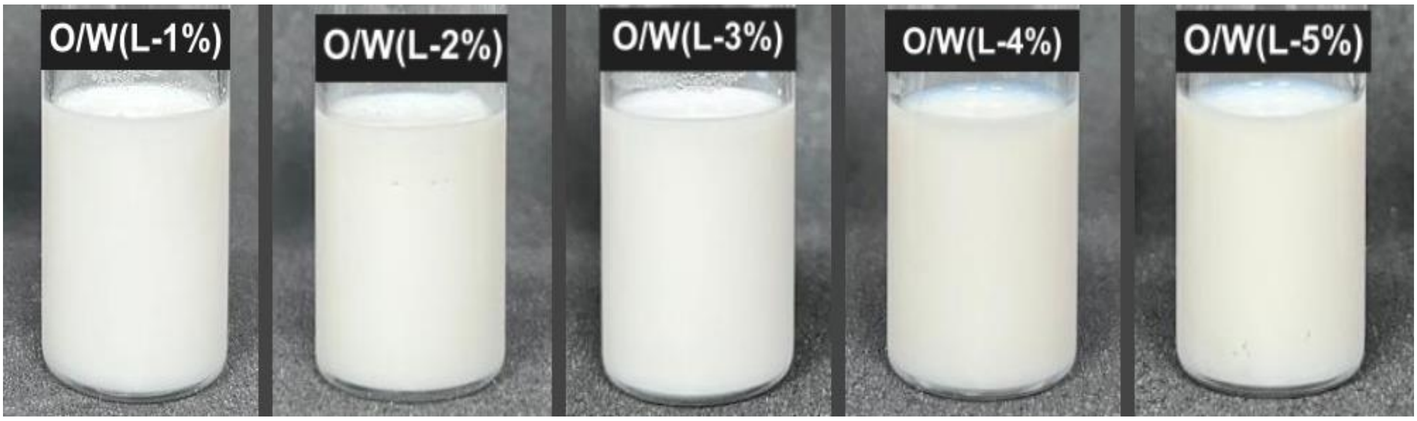

- **O/W(L-1%)** - Pre-emulsion (8.2% w/w) containing 1% (w/w) lecithin (composition: 0.25 g lecithin : 2 g linseed oil : 25 g de Milli-Q water);
- **O/W(L-2%)** - Pre-emulsion (9.1% w/w) containing 2% (w/w) lecithin (composition: 0.5 g lecithin : 2 g linseed oil : 25 g de Milli-Q water);
- **O/W(L-3%)** - Pre-emulsion (9.9% w/w) containing 3% (w/w) lecithin (composition: 0.75 g lecithin : 2 g linseed oil : 25 g de Milli-Q water);
- **O/W(L-4%)** - Pre-emulsion (10.7% w/w) containing 4% (w/w) lecithin (composition: 1.0 g lecithin : 2 g linseed oil : 25 g de Milli-Q water);
- **O/W(L-5%)** - Pre-emulsion (11.5% w/w) containing 5% (w/w) lecithin (composition: 1.25 g lecithin : 2 g linseed oil : 25 g de Milli-Q water).

Figure S3: Linear regression analysis was used to determine the IC_{50} values for the antioxidant activity of ophthalmic nanoformulations, based on DPPH radical scavenging: (a) OphtNE-3.70% and (b) OphtNE-3.66%(K1%).

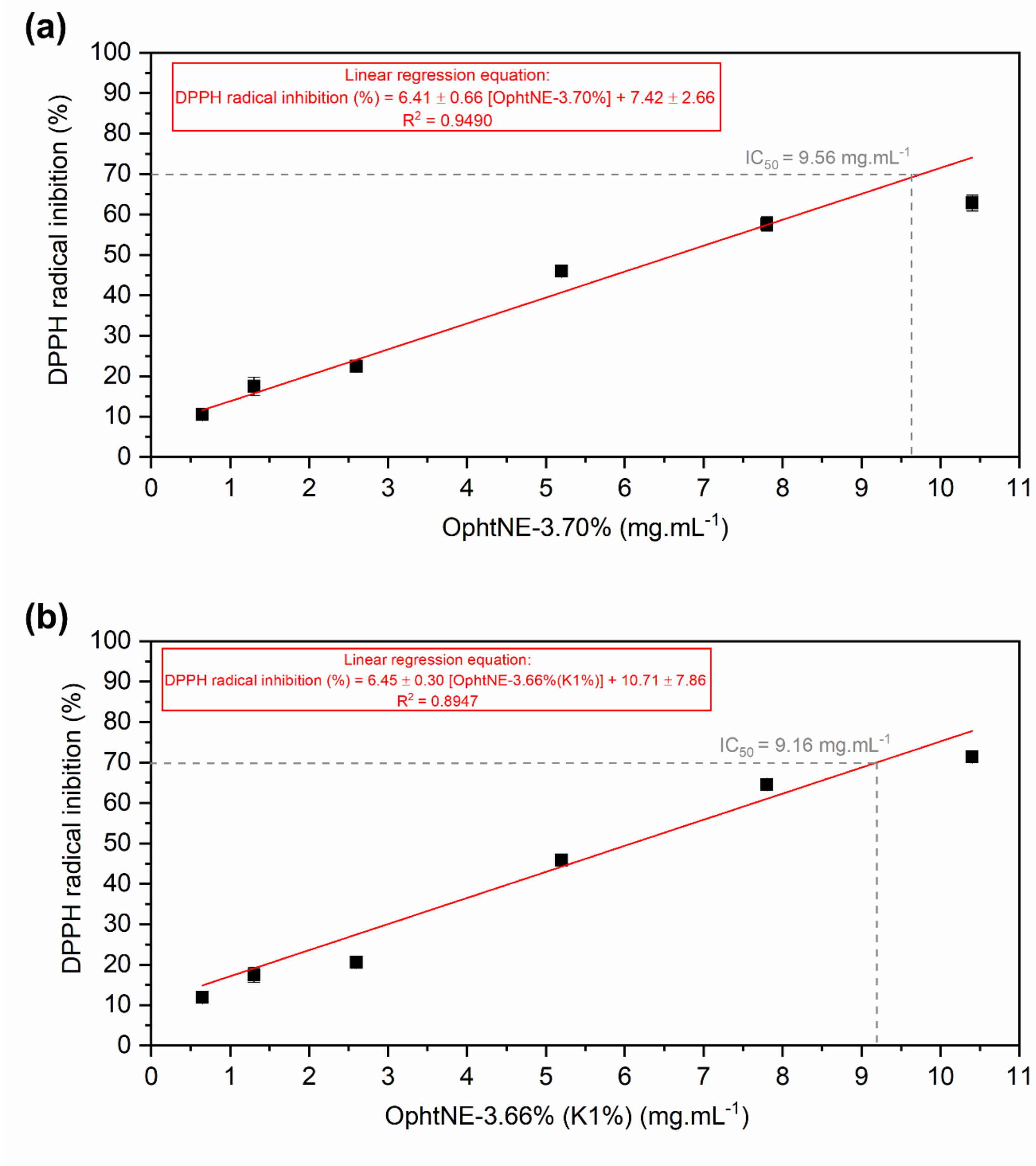

SUPPLEMENTARY ANOVA RESULTS

Table S3: Two-way ANOVA evaluating the effects of treatment, concentration, and their interaction on MTT assay absorbance in L929 cells at 24 and 72 h. This analysis was conducted to further investigate the statistically significant interaction between treatment and concentration identified in the three-way ANOVA (Table 4 of the paper). Reported values include degrees of freedom (DF), F-statistics, *p*-values, generalized eta-squared (GE), and adjusted *p*-values. Significant effects are indicated for $p < 0.05$.

Time (h)	Effect	DF_n	DF_d	*F*	*p*	ges	*p*_adjust	$p < 0.05$
24	Treatment	1	99	57.985	$1.59\ 10^{-11}$	0.369	$3.18\ 10^{-11}$	*
24	Concentration ($mg{\cdot}mL^{-1}$)	8	99	22.881	$2.00\ 10^{-19}$	0.649	$4.00\ 10^{-19}$	*
24	Treatment:Concentration	8	99	5.244	$1.76\ 10^{-5}$	0.298	$3.52\ 10^{-5}$	*
72	Treatment	1	99	27.365	$9.42\ 10^{-7}$	0.217	$1.88\ 10^{-3}$	*
72	Concentration ($mg{\cdot}mL^{-1}$)	8	99	39.913	$1.08\ 10^{-27}$	0.763	$2.16\ 10^{-27}$	*
72	Treatment:Concentration	8	99	4.287	0.000186	0.257	0.000372	*

* significant ($p < 0.05$)

Table S4: One-way ANOVA assessing the effect of concentration at each exposure time (24 and 72 h) by pooling treatment groups, based on the significant Treatment:Concentration interaction observed in the two-way ANOVA (Table S3). Values reported include degrees of freedom (DF), F-statistics, *p*-values, generalized eta-squared (ges), and adjusted *p*-values. Significant effects are indicated for $p < 0.05$.

Concentration ($mg{\cdot}mL^{-1}$)	Time (h)	DF_n	DF_d	*F*	*p*	ges	*p*_adjust	$p < 0.05$
Negative Control	24	1	99	0	1	0	1	ns
Positive Control	24	1	99	0	1	0	1	ns
0.65	24	1	99	1.611	0.207	0.016	0.414	ns
1.30	24	1	99	2.359	0.128	0.023	0.256	ns
2.60	24	1	99	8.917	0.004	0.083	0.008	*
5.20	24	1	99	30.559	$2.63\ 10^{-7}$	0.236	$5.26\ 10^{-7}$	*
7.80	24	1	99	43.988	$1.76\ 10^{-9}$	0.308	$3.52\ 10^{-9}$	*
10.4	24	1	99	10.622	0.002	0.097	0.004	*
13.0	24	1	99	1.879	0.174	0.019	0.348	ns
Negative Control	72	1	99	0	1	0	1	ns
Positive Control	72	1	99	0	1	0	1	ns
0.65	72	1	99	0.136	0.714	0.001	1	ns
1.30	72	1	99	5.225	0.024	0.05	0.048	*
2.60	72	1	99	11.551	0.000977	0.104	0.001954	*
5.20	72	1	99	27.421	$9.21\ 10^{-7}$	0.217	$1.84{\cdot}10^{-3}$	*
7.80	72	1	99	17.234	$7.01\ 10^{-5}$	0.148	0.0001402	*
10.4	72	1	99	0.035	0.852	0.000353	1	ns
13.0	72	1	99	0.059	0.809	0.000591	1	ns

* significant ($p < 0.05$); ns: not significant

Table S5: Two-way ANOVA evaluating the effects of concentration, time, and their interaction on MTT assay absorbance in L929 cells for each treatment group (OphtNE-3.70% and OphtNE-3.66%(K1%)). This analysis was conducted to further investigate the statistically significant Concentration:Time interaction identified in the three-way ANOVA (Table 4 of the paper). Reported values include degrees of freedom (DF), F-statistics, *p*-values, generalized eta-squared (ges), and adjusted *p*-values. Significant effects are indicated for $p < 0.05$.

Treatment	Effect	DF_n	DF_d	*F*	*p*	ges	*p*_adjust	$p < 0.05$
OphtNE-3.70%	Concentration ($mg·mL^{-1}$)	8	99	35.245	$1.01·10^{-25}$	0.74	$2.02·10^{-25}$	*
OphtNE-3.70%	Time (h)	1	99	1.316	0.254	0.013	0.508	ns
OphtNE-3.70%	Concentration:Time	8	99	2.737	0.009	0.181	0.018	*
OphtNE-3.66%(K1%)	Concentration ($mg·mL^{-1}$)	8	99	33.36	$7.15·10^{-25}$	0.729	$1.43·10^{-24}$	*
OphtNE-3.66%(K1%)	Time (h)	1	99	0.476	0.492	0.005	0.984	ns
OphtNE-3.66%(K1%)	Concentration:Time	8	99	0.983	0.454	0.074	0.908	ns

* significant ($p < 0.05$); ns: not significant

Table S6: One-way ANOVA assessing the effect of concentration for OphtNE-3.70%, pooling exposure times, based on the significant Concentration × Time interaction observed in the two-way ANOVA (Table S5). Values reported include degrees of freedom (DF), F-statistics, *p*-values, generalized eta-squared (ges), and adjusted *p*-values. Significant effects are indicated for $p < 0.05$.

Treatment	Concentration ($mg·mL^{-1}$)	DF_n	DF_d	*F*	*p*	ges	*p*_adjust	$p < 0.05$
OphtNE-3.70%	Negative Control	1	99	2.772	0.099	0.027	0.198	ns
OphtNE-3.70%	Positive Control	1	99	0.000161	0.99	$1.63·10^{-6}$	1	ns
OphtNE-3.70%	0.65	1	99	0.829	0.365	0.008	0.73	ns
OphtNE-3.70%	1.30	1	99	2.225	0.139	0.022	0.278	ns
OphtNE-3.70%	2.60	1	99	0.912	0.342	0.009	0.684	ns
OphtNE-3.70%	5.20	1	99	0.007	0.935	$6.67·10^{-5}$	1	ns
OphtNE-3.70%	7.80	1	99	6.19	0.015	0.059	0.03	*
OphtNE-3.70%	10.4	1	99	9.288	0.003	0.086	0.006	*
OphtNE-3.70%	13.0	1	99	0.99	0.322	0.01	0.644	ns

* significant ($p < 0.05$); ns: not significant
